# ICA-S^3^M: switching state-space model guided automatic EEG artifact removal from independent components

**DOI:** 10.3389/fnins.2026.1865301

**Published:** 2026-07-31

**Authors:** Si Long Jenny Tou, Mingjian He, Scott Yeiichi Oshiro, Proloy Das, Patrick L. Purdon

**Affiliations:** 1 Department of Anesthesiology, Stanford Medicine, Palo Alto, CA, United States; 2 Department of Psychology, Stanford University, Palo Alto, CA, United States; 3 TCG CREST, Kolkata, India; 4 Department of Bioengineering, Stanford University, Palo Alto, CA, United States

**Keywords:** artifact correction, Bayesian switching model, EEG artifact removal, electromyographic contamination, EMG artifact, independent component analysis, muscle artifact, oscillator model

## Abstract

**Background:**

Electromyographic (EMG) artifact is among the most challenging contaminants in scalp EEG: its broadband spectrum overlaps directly with neural activity, and sustained muscle contractions distribute across many independent components (ICs) rather than segregating cleanly—an effect we term EMG smearing.

**Methods:**

We propose ICA-S^3^M, a two-stage pipeline that first decomposes the recording via independent component analysis (ICA), then applies a switching state-space model to each retained IC. This model separates neural oscillations, modeled as damped oscillators fitted to the recording's own spectral content, from broadband artifact, modeled as an autoregressive (AR(2)) process. The method produces an explicit artifact probability at every time point and requires no training data. We evaluated ICA-S^3^M against a CNN from EEGdenoiseNet in three settings: simulated EEG with known ground truth; a semi-synthetic real-EEG dataset constructed from 29 participants through 11,025 expert IC–trial classifications, in which a trained rater classified every IC on a per-trial basis across interleaved rest and facial-movement segments; and a naturalistic recording of musical improvisation.

**Results:**

In simulation, ICA-S^3^M reduced RRMSE from 0.83 to 0.23 and improved SNR by 12.4 dB, versus 1.2 dB for the CNN. On the semi-synthetic dataset, ICA-S^3^M significantly outperformed the CNN in 38 of 42 tested scenarios spanning seven metrics, two time periods, and three scalp regions, while leaving clean segments essentially untouched. Critically, the CNN exhibited an “alpha hallucination” failure mode, injecting spurious alpha-band peaks where none exist in the ground truth; we reproduced this with an alpha-free control simulation and observed it again on real scalp recordings.

**Discussion:**

ICA-S^3^M avoids this failure by adapting to each recording's own oscillatory structure rather than imposing learned spectral templates. The method offers a principled, interpretable, and training-free alternative for EMG artifact removal in challenging EEG recordings.

## 1 Introduction

Electroencephalography (EEG) provides a non-invasive measure of neural dynamics with millisecond temporal resolution, but recorded signals are often contaminated by non-neural artifacts (Urigüen and Garcia-Zapirain, 2015; Radüntz et al., 2017). Sources of contamination include electrooculographic (EOG) activity from eye movements and blinks, electrocardiographic (ECG) signals, electrode drift caused by movement, and electromyographic (EMG) activity from facial and cervical muscles (Urigüen and Garcia-Zapirain, 2015; Gorjan et al., 2022). Among these, EMG artifact poses the greatest challenge (Urigüen and Garcia-Zapirain, 2015). First, the signal-to-noise ratio (SNR) of the underlying neural signal relative to EMG can be extremely low, and EMG artifacts often do not have a stable, defined topology (Muthukumaraswamy, 2013; Mumtaz et al., 2021). Second, EMG activity has a broadband spectral signature extending from below 10Hz to beyond 200Hz, overlapping substantially with the physiologically meaningful EEG frequency range of approximately 0.5–35Hz (Muthukumaraswamy, 2013; McMenamin et al., 2010). This spectral overlap makes it fundamentally difficult to separate EMG from neural signal using frequency-domain filtering alone. These issues are particularly problematic in clinical populations where movement cannot be controlled, such as intensive care unit (ICU) monitoring Hwang et al. (2022), intraoperative neurophysiology (Purdon et al., 2015, 2013; Mukamel et al., 2014), and pediatric EEG, as well as in emerging paradigms involving speech production Janssen et al. (2020); Porcaro et al. (2015), mobile EEG Gorjan et al. (2022), and brain-computer interfaces (BCI) (Kaya, 2019).

The most common approaches to managing EEG artifacts are prevention and rejection (Urigüen and Garcia-Zapirain, 2015; Kaya, 2019; Zhang et al., 2024). Prevention strategies aim to minimize artifact at the point of data collection through experimental design choices such as having participants sit still and providing explicit instructions not to move (Gorjan et al., 2022), as well as through hardware improvements including active electrodes with built-in preamplifiers (Xu et al., 2017) or disk electrodes (Young et al., 2006). While prevention is highly effective, it is not always feasible, again in clinical settings where movement or naturalistic behavior are intrinsic (Urigüen and Garcia-Zapirain, 2015; Hwang et al., 2022; Purdon et al., 2015, 2013; Mukamel et al., 2014; Janssen et al., 2020; Porcaro et al., 2015; Gorjan et al., 2022; Kaya, 2019). When significant artifact contamination is present despite preventive measures, the affected data are typically discarded. Rejection methods range from simple amplitude-based thresholding (e.g., excluding epochs exceeding ±150 μV) (Ledwidge et al., 2025; Delussi et al., 2020) to more sophisticated approaches employing statistics and deep learning for automated segment or channel rejection (Brodbeck et al., 2023; Oliveira et al., 2017; Jas et al., 2017). However, rejection is inherently lossy: discarding contaminated segments reduces statistical power, and in severe cases, such as sustained muscle tension throughout a recording, rejection may leave too little data for meaningful analysis.

A more desirable alternative is to separate and extract the artifact component from the EEG, recovering the underlying clean neural signal. The dominant approach in the literature for achieving this is independent component analysis (ICA) (Makeig et al., 1995; Jung et al., 2000; Delorme and Makeig, 2004; Gorjan et al., 2022). In brief, ICA decomposes multichannel EEG into a set of temporally independent components (ICs), each associated with a spatial topography (the mixing matrix column) and a time course. Under the assumption that neural sources and noise sources are generated independently, artifact activity should concentrate in distinct ICs that can be identified and removed (Jung et al., 2000). Identification of artifact ICs can be performed through manual inspection of each component's time course, power spectrum, and scalp topography (Jung et al., 2000), or through automated classification using trained models such as ICLabel (Pion-Tonachini et al., 2019) among many others (Winkler et al., 2011; Mognon et al., 2011; Radüntz et al., 2017; Gabard-Durnam et al., 2018). For phasic artifacts with stereotyped spatial signatures, such as eye blinks, saccades, and cardiac activity, ICA-based removal is generally effective, as these sources tend to concentrate in one or a small number of ICs with distinctive topographies (Jung et al., 2000; Li et al., 2006).

In practice, however, ICA performs poorly for sustained, time-varying, and spatially distributed EMG contamination (McMenamin et al., 2010; Muthukumaraswamy, 2013). We highlight two principal reasons for this failure. First, ICA is constrained by rank deficiency. The decomposition can recover at most as many independent sources as there are recording channels. High-density EMG contamination, for example, from the temporalis, frontalis, or cervical muscles, involves many independent myogenic sources, potentially dozens of motor unit action potentials firing asynchronously across multiple muscle groups. With typical EEG montages of 32 or 64 channels, ICA lacks sufficient degrees of freedom to isolate all such sources (Mumtaz et al., 2021; McMenamin et al., 2010; Delorme et al., 2012). Even with high-density montages of 128 or 256 channels, the number of independent myogenic generators during sustained contraction can exceed the available dimensionality. Second, ICA assumes a stationary mixing matrix, that is, a fixed spatial relationship between sources and sensors throughout the recording. If muscle activity patterns change over time (e.g., a participant gradually tenses, intermittent bruxism occurs, or different muscle groups are engaged at different points), the spatial topography of EMG contamination shifts, violating the stationarity assumption and degrading decomposition quality (Urigüen and Garcia-Zapirain, 2015; Delorme et al., 2012).

The direct consequence of these two limitations is what we will refer to as the *EMG smearing effect*: rather than concentrating in a small number of clearly artifactual ICs, tonic and time-varying EMG activity distributes across many components, each containing a mixture of neural and muscular activity. This creates a fundamental dilemma: removing all contaminated components discards too much neural signal, but retaining them preserves artifacts. Automated classification tools such as ICLabel, which were designed to categorize ICs into discrete source classes, cannot resolve this ambiguity when a single IC contains contributions from both neural and myogenic sources. The EMG smearing effect has been widely recognized in the literature as a key limitation of ICA-based artifact removal for EMG (McMenamin et al., 2010; Fitzgibbon et al., 2015; Muthukumaraswamy, 2013). An ideal method would operate within each mixed IC to separate neural from myogenic content on the basis of their distinct spectral and temporal dynamics, rather than requiring clean spatial separation at the ICA stage.

Several alternative approaches to EMG artifact removal have been explored beyond ICA. Canonical correlation analysis (CCA) exploits differences in autocorrelation structure between EEG and EMG to separate the two, but assumes stationarity and requires careful parameter selection (De Clercq et al., 2006). Empirical mode decomposition (EMD) and wavelet-based methods offer time-frequency flexibility but lack principled statistical frameworks for distinguishing spectrally overlapping sources (Safieddine et al., 2012). More recently, deep learning approaches have shown promise (Raj et al., 2025): GAN-based architectures such as AnEEG (Kalita et al., 2024), encoder-decoder models such as EEGDNet (Pu et al., 2022), and hybrid approaches that apply deep networks to ICA-decomposed components such as IC-U-Net (Chuang et al., 2022). However, these methods require large labeled (Chuang et al., 2022) or semi-simulated training datasets (Pu et al., 2022), and their generalizability across recording setups, electrode montages, and artifact types remains uncertain (Raj et al., 2025). Critically, none of these approaches leverages parametric models of neural oscillatory dynamics to distinguish neural signal from broadband artifact at the level of individual components. Without an appropriate, interpretable model for neural signals in the EEG time series data, the effectiveness of removing artifacts often comes at a cost of losing real electrophysiological signals of interest. Previously, we have described how switching state space models (SSSM) featuring distinct model components for noise and EEG oscillations, respectively, can be used to separate neural signals from noise (Stone et al., 2025). We found that the distinct models for signal and noise could both “protect” neural signals from being misidentified as noise, but also constrain artifact activity as being less well explained by neural signal models. A limitation of the method was that it operated on scalar time series and thus could not benefit from pooling spatial information about signals or artifacts across channels.

In this manuscript, we propose ICA-S^3^M, a two-stage pipeline that benefits from ICA's highly effective spatial decompositions while addressing the EMG smearing problem using switching state-space models. The method is inherently interpretable at two levels– mechanistically, by incorporating a flexible generative model for the underlying neural signals and noise, and probabilistically, since the model defines uncertainty at multiple levels to provide posterior probabilities of artifact states as well as the component neural and artifact signals. In the first stage, ICA is applied in the conventional manner to separate sources that are spatially and temporally distinct, such as eye blinks, cardiac activity, and major neural source regions. In the second stage, each retained IC is processed individually using a switching state-space model (SSSM) based on the framework proposed by He et al. (2023). Within this framework, neural oscillatory content (Urigüen and Garcia-Zapirain, 2015) is modeled using damped oscillator state-space models (Matsuda and Komaki, 2017), while EMG artifact is modeled as a broadband autoregressive AR(2) process (Lawhern et al., 2013). A discrete Markov process governs the transition between clean and artifact states at each time point, allowing the model to adapt to the time-varying presence and absence of EMG contamination within each IC. By operating within each IC rather than across channels and considering temporal dynamics explicitly, ICA-S^3^M effectively sidesteps the rank deficiency problem: even when EMG is smeared across many ICs, the within-IC state-space model can separate the oscillatory and broadband components of each mixture based on their distinct dynamics. Additionally, the oscillation decomposition step (He, 2024) boosts quality of separation in the contaminated segments, by providing informed priors using data-driven characterization of the neural content within clean segments, for the final artifact separation.

The main contributions of this work are threefold: (1) we introduce ICA-S^3^M, a two-stage pipeline that addresses the EMG smearing effect by combining ICA decomposition with within-component switching state-space modeling, enabling separation of neural oscillatory content from broadband artifact even when the two are mixed within individual ICs; (2) we leverage data-driven oscillation decomposition to characterize the spectral structure of clean segments, providing informed priors for artifact separation without relying on externally trained models or labeled datasets; and (3) we provide a comprehensive evaluation across three complementary settings, (1) simulated EEG with known ground truth, (2) a semi-synthetic dataset consisting interleaved rest and artifact periods from a facial movement paradigm, and (3) a naturalistic recording during musical performance. These three evaluations benchmark ICA-S^3^M against a state-of-the-art supervised deep learning baseline (Complex CNN). Across all three settings, ICA-S^3^M demonstrated superior signal reconstruction fidelity and spectral preservation compared to CNN, while requiring no training data.

The remainder of this paper is organized as follows: Section 2 describes the ICA-S^3^M model, datasets, preprocessing, and evaluation metrics; Section 3 presents results from the simulation, semi-synthetic real data, and naturalistic evaluations; and Section 4 discusses the implications, limitations, and future directions of the proposed approach.

## 2 Methods

### 2.1 Overview

**Figure 1:**
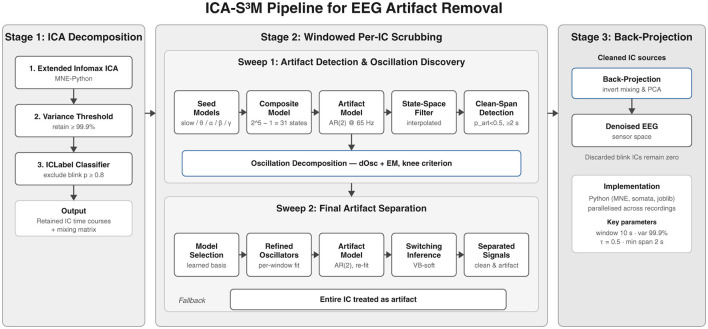
Overview of the ICA-S^3^M pipeline for EEG artifact removal. Stage 1 performs ICA decomposition via Extended Infomax, retaining components that pass variance and ICLabel thresholds. Stage 2 applies a two-sweep, windowed artifact scrubbing procedure using switching state-space models (SOMATA): Sweep 1 discovers oscillatory structure and identifies clean spans, while Sweep 2 uses refined models to produce final clean/artifact separation per IC. Stage 3 back-projects the separated components to sensor space, yielding denoised EEG and isolated artifacts.

ICA-S^3^M is a two-stage pipeline for automatic artifact removal from continuous EEG recordings. In the first stage, the full recording is decomposed into independent components (ICs) via extended Infomax ICA. In the second stage, each retained IC is processed independently within non-overlapping temporal windows using a switching state-space model (SSSM) that separates neural oscillatory activity from artifact contamination. Cleaned IC time courses are then back-projected to sensor space to yield the denoised EEG. The overall pipeline is illustrated in Figure 1. Pseudo code is in Algorithm 1. Key parameters are listed in Table 1. A per-component ablation that runs each stage in isolation, including ICA-only (ICLabel-based component rejection) and SSSM-only (single-channel state-space scrubbing applied directly to sensor signals without the ICA decomposition), is reported in Appendix 4.0.0.5. ICA-S^3^M significantly outperforms both alternatives on artifact-period RRMSE, SNR improvement, and waveform correlation across frontal, temporal, and occipital regions.

Algorithm 1 ICA-S^3^M artifact removal: whole-recording ICA, then per-component windowed switching state-space scrubbing. 
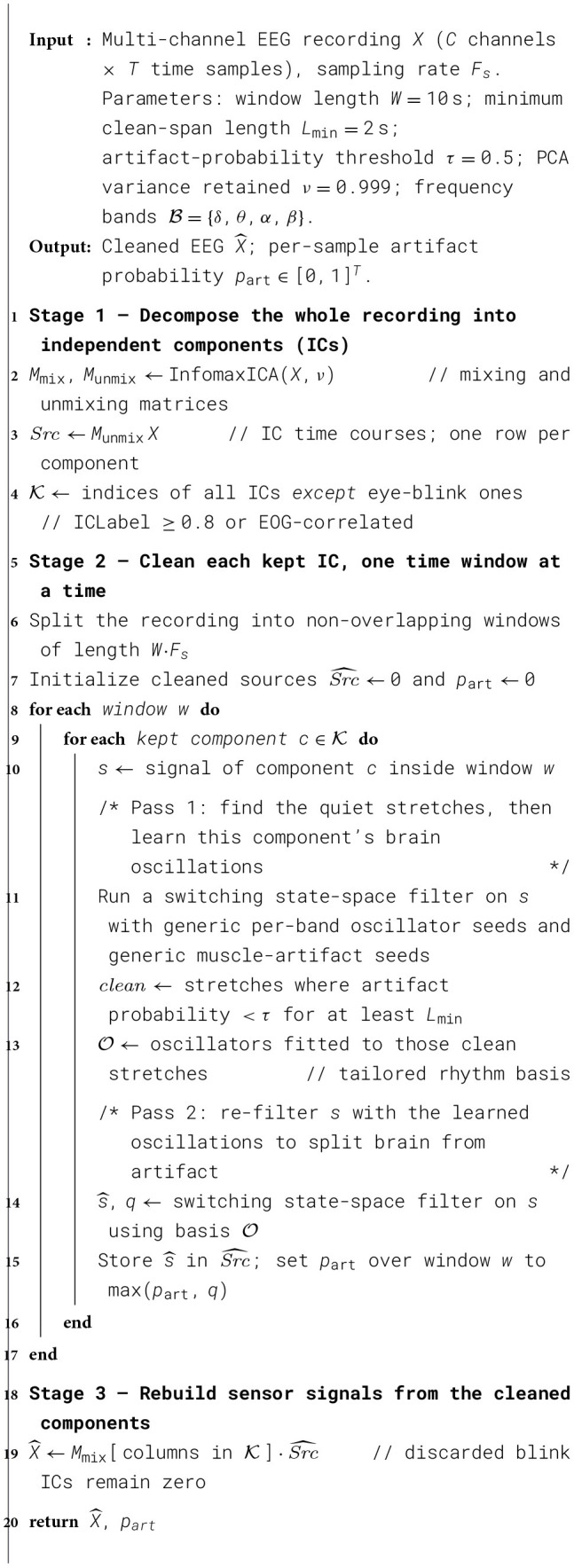

**Table 1:** Key pipeline parameters and their default values.

Parameter	Default	Description
Variance explained	0.999	ICA component selection threshold
Window length	10.0 s	Non-overlapping window duration
Minimum clean span	2.0 s	Minimum duration for dOsc fitting
Clean-span clean fraction	0.9	Minimum fraction of clean samples within a qualifying span
*R* sub-window length	1.0 s	Sub-window for per-window observation-noise estimator
*R* sub-window hop	0.5 s	Hop between successive sub-windows
*R* quantile	0.10	Per-window *R* taken as this quantile of per-sub-window estimates

### 2.2 ICA-S^3^M model

#### 2.2.1 Stage 1: ICA decomposition

Extended Infomax ICA (Lee et al., 1999) was applied to the full continuous EEG recording using MNE-Python (Gramfort et al., 2013). The number of independent components was determined by a variance-explained threshold, retaining the minimum number of components necessary to account for at least 99.9% of the total variance. Details on number of ICs retained is in Appendix 4.1. The IC source time courses and columns of the mixing matrix were extracted for per-component state-space processing.

#### 2.2.2 Stage 2: windowed per-IC artifact scrubbing

Each retained IC source was segmented into non-overlapping windows of 10s duration. Within each window, artifact scrubbing proceeded through two sequential sweeps: an artifact detection and oscillation discovery sweep, followed by a refined artifact and neural oscillatory signals separation sweep. All state-space modeling was performed using the SOMATA library (He, 2024).

##### 2.2.2.1 Sweep 1: artifact detection and neural oscillation discovery

The goal of the first sweep was to identify time points contaminated by artifacts and to characterize the oscillatory content of clean segments, providing informed priors for the second sweep. The sweep consisted of the following steps:

**Seed model construction**. For each of four canonical frequency bands, including slow (1 Hz), theta (5 Hz), alpha (10 Hz), and beta (20 Hz), an oscillator model (Osc) (He et al., 2023; Matsuda and Komaki, 2017) was constructed as a second-order state-space model with complex-conjugate poles defined by a center frequency *f*_*k*_ and damping coefficient *a*_*k*_ (Table 2). The driving noise variance $\sigma_{k}^{2}$ of each oscillator was scaled such that the peak of the model's theoretical power spectral density (PSD) matched the corresponding peak in the empirical multitaper PSD of the IC window data, estimated using multitaper spectral analysis in SOMATA (He, 2024).
**Composite model construction**. All non-empty subsets of the four band-specific oscillator models were enumerated, yielding 2^4^−1 = 15 composite models representing every possible combination of oscillatory activity. Each composite model served as a candidate “clean” state-space model.
**Artifact model**. Artifact activity was modeled as a second-order autoregressive [AR(2)] process with a complex-conjugate pole pair at 65 Hz and damping *a* = 0.5, capturing broadband and high-frequency artifact characteristics. The AR(2) artifact model is physiologically grounded rather than ad hoc. Surface EMG arises from the spatio-temporal summation of biphasic motor-unit action potentials (MUAPs), each of which reflects the resistive–capacitive charge–discharge dynamics of the neuromuscular junction and the depolarization–repolarization cycle of the muscle-fiber membrane (Basmajian and De Luca, 1985; De Luca, 1997; Farina et al., 2004). These dynamics are well-approximated by a damped second-order linear system, which is mathematically equivalent to an AR(2) process and admits a single dominant resonance whose center frequency is determined by the membrane time constants and conduction velocity. We set the resonance to 65 Hz to match the dominant peak typically observed in surface-EMG power spectra (≈50–150 Hz; Merletti and Parker, 2004), with the damping ratio left as a free parameter fit per window.
The driving noise variance of the base model was matched to the empirical PSD peak in the alpha band. To accommodate varying artifact amplitudes, the base artifact model was replicated at four state noise covariance scales (0.2, 1, 5, and 25 × the base variance), yielding four artifact models spanning a wide range of artifact signal-to-noise ratios. Each composite clean model was then paired with each of the four scaled artifact models in the switching state-space framework, allowing the inference to select the artifact amplitude that best explains the observed data at each time point.
**Observation noise**. The observation noise variance *R* was estimated from the IC window data using a high-frequency cutoff estimator and assigned uniformly to all candidate models. Bursts of EMG activity can inflate a single full-window estimate of the variance. *R* was therefore obtained as the 10th percentile of per-sub-window estimates: a 1.0 s sub-window was slid across the 10 s IC window in 0.5 s steps, the high-frequency-cutoff estimator (cutoff *F*_*s*_/2 − 5 Hz) was applied to each sub-window, and the quantile across the resulting set was taken as the window's *R*. This recovers a noise floor representative of the truly quiet stretches of the window rather than one inflated by transient artifact energy.
**Switching state-space inference**. The artifact detection was performed using a switching state-space model whose discrete states are the composite states (*i, j*), pairing candidate oscillator model *i* with a scaled copy of the AR(2) EMG model at variance level $\sigma_{\text{art},j}^{2}\in \left\{0,0.2,1.0,5.0,25.0\right\}$, where $\sigma_{\text{art},j}^{2}=0$ denotes the pure-oscillator variant with no artifact component. Transition probabilities between composite states were specified by the artifact switching design, and the interpolated-method inference returned the posterior $M_{\text{prob}}^{\left(i,j\right)}\left(t\right)$ over all composite states at every time point (He et al., 2023).
**Artifact score**. Rather than thresholding a binary “clean vs. artifact” posterior directly, we assigned each composite (*i, j*) a fractional artifact weight

$$\begin{array}{l} w_{ij}=\frac{\sigma_{\text{art},j}^{2}}{\sigma_{\text{art},j}^{2}+1}, \end{array}$$ (1)

equal to the fraction of instantaneous signal variance that the AR(2) EMG component contributes within that composite (under unit oscillator variance) (He et al., 2023). Pure-oscillator composites therefore carry *w* = 0, variance-matched composites carry *w* = 0.5, and composites dominated by a high-variance AR(2) component carry *w* approaching 1 (specifically ≈0.17, 0.50, 0.83, 0.96 for the four non-zero scales). The per-time-point artifact score is then the posterior-expected artifact-variance fraction,


$$\begin{array}{l} p_{\text{art}}\left(t\right)=\underset{i,j}{\sum }M_{\text{prob}}^{\left(i,j\right)}\left(t\right)w_{ij}\in \left[0,1\right]. \end{array}$$ (2)

**Table 2:** Canonical frequency band definitions used for oscillator model construction.

Band	Center frequency (Hz)	Half-bandwidth (Hz)	Damping (*a*)	Coverage (Hz)
Slow	1.5	1.5	0.99	0–3
Theta	5.25	2.25	0.95	3–7.5
Alpha	10.25	2.75	0.95	7.5–13
Beta	21.5	8.5	0.95	13–30
Gamma	40.0	10.0	0.95	30–50

This weighting is important because, in the EMG smearing regime, many high-posterior composites contain *both* an oscillator and an AR(2) component; a naive rule that labeled any artifact-containing composite as “100% artifact” would therefore miss precisely the mixed regime that motivates our approach. The variance-fraction weighting instead charges posterior mass on such composites against the clean label in proportion to how much of the composite's variance is EMG-driven.

**Clean span identification**. Time points with *p*_art_(*t*) < 0.5 were labeled clean. Contiguous spans of at least 2 s containing at least 90% clean samples were then identified, and each candidate span was edge-trimmed inward so that its first and last samples were both clean—non-clean (“outlier”) samples are permitted only in the span interior. After edge-trimming, any span shorter than the 2 s minimum was discarded; the remaining spans were retained for oscillation decomposition. This rule prevents the dOsc fit from being seeded with sub-second artifact tails at span boundaries while still tolerating brief mid-span excursions in long segments with few artifacts.
**Oscillation decomposition**. In order to find the EEG oscillations in each qualifying clean span, a Decomposed Oscillator Model (dOsc) (He, 2024) was fitted using expectation-maximization (EM) with up to 50 iterations and a pre-iteration initialization phase. The model search considered up to 7 oscillatory components, and the final model order was selected via a “knee” criterion applied to the sequence of model evidence values. For each identified oscillatory component, the estimated parameters, includig damping coefficient *a*, center frequency *f*, and driving noise variance σ^2^, were assigned to the nearest canonical frequency band.

##### 2.2.2.2 Sweep 2: final artifact separation

The second sweep used the oscillation parameters discovered in Sweep 1 to construct refined state-space models and perform the definitive clean/artifact separation.

**Model selection**. Oscillator parameters for the refined models were selected according to the following priority: (1) dOsc parameters extracted from the current window, summarized as the median *a*, *f*, and σ^2^ per frequency band across all decomposed spans; (2) dOsc parameters from the nearest preceding window, if the current window yielded no valid decompositions; (3) PSD-seed parameters from Sweep 1 as a fallback. If no oscillator parameters were available from any source, the entire window was conservatively marked as artifact.
**Refined oscillator models**. For each frequency band with available parameters, a single oscillator model was constructed using the median damping, frequency, and driving noise values across all dOsc triples assigned to that band.
**Artifact model**. The Sweep 2 artifact model was an AR(2) process with a complex-conjugate pole pair at 65 Hz and damping *a* = 0.5, with driving noise variance matched to the empirical PSD at 65 Hz.
**Switching inference**. The interpolated method is used for inference for computational efficiency in our implementation, but variational bayes methods are available. The output of this sweep is comprised of the cleaned IC signal, the separated artifact IC signal, and a per-time-point artifact probability vector for each window.
**Residual spike removal**. Residual transient spike artifacts that were not absorbed by the AR(2) artifact model were flagged and removed. Samples in the “cleaned” IC whose absolute value exceeded ten times the median absolute deviation (MAD) were defined as spike peaks, and each contiguous peaks were expanded outward to the nearest zero crossings of the signal; overlapping regions sharing zero-crossing boundaries were merged. Within each spike region, a first-order autoregressive [AR(1)] model was fitted via Yule–Walker to the original “cleaned” IC samples, and the artifact switching model (Stone et al., 2025) was re-run locally on the spike region using the fitted AR(1) as the sole artifact model. Clean oscillator models from Sweep 2 containing any component with center frequency below 3 Hz were excluded from this pass to prevent the slow oscillator from absorbing the sharp transient. The new cleaned signal replaced the output within th spike region.

#### 2.2.3 Stage 3: back-projection to sensor space

After processing all windows for all retained ICs, the cleaned and artifact IC time courses were back-projected to sensor space using the ICA mixing matrix (restricted to the columns corresponding to retained components). The PCA mean and pre-whitening transformations applied during ICA were inverted to recover the original sensor-space scale.

#### 2.2.4 Implementation

The pipeline was implemented in Python using MNE-Python (Gramfort et al., 2013) for ICA decomposition, data I/O, and channel handling, and the SOMATA library (He, 2024) for all state-space modeling, including oscillator and autoregressive model construction, the DecomposedOscillatorModel, the ArtifactScrubber, switching design specification, multitaper spectral estimation, and observation noise estimation. Batch processing across multiple recordings was parallelized at the file level using joblib package.

### 2.3 Data

#### 2.3.1 Simulation study

To evaluate artifact removal performance with known ground truth, we generated 60s of realistic multi-channel EEG using the MNE-Python forward modeling (Gramfort et al., 2013). Scalp-level signals were projected from 14 cortical source regions selected from the Destrieux atlas (aparc.a2009s parcellation) (Destrieux et al., 2010), spanning bilateral auditory cortex (superior temporal gyrus, transverse temporal gyrus, and planum temporale), inferior frontal gyrus (opercular and triangular parts), mid-anterior and posterior-dorsal cingulate cortex, inferior parietal lobule (supramarginal gyrus), and precuneus. Source time courses were constructed by superimposing a damped harmonic oscillators driven by Gaussian white noise (Matsuda and Komaki, 2017). Each cortical region was assigned physiologically motivated frequency bands: theta (4–7 Hz) for temporal and frontal regions, alpha (8–12 Hz) for parietal, cingulate, and precuneus regions, beta (15–30 Hz) for frontal and parietal regions, and low gamma (30–50 Hz) for auditory cortex. Oscillator parameters (damping coefficient *a* ∈ [0.9, 0.99], center frequency drawn uniformly within band limits) were randomized per bout, and the driving noise variance σ^2^ was calibrated to achieve a target RMS amplitude of 10 nAm, scaled by band-specific magnitude factors (5–20 × for alpha, 2–10 × for beta). Each band–region pair generated 5–7 bouts of 5–30 s duration at random onset times, with 1 s raised-cosine fade-in/fade-out envelopes to avoid spectral artifacts from sharp transients. Source time courses were projected to 59 EEG sensors using the MNE sample dataset forward solution (BEM model, oct-6 source space). The resulting clean EEG was stored at the mne forward model native sampling rate of 600.6 Hz.

Two types of artifacts were injected into the clean EEG to simulate realistic contamination scenarios:

##### 2.3.1.1 EMG artifacts

Five EMG artifact segments were generated using second-order autoregressive [AR(2)] processes. For each segment, a seed electrode was selected with probability weighted 4 × toward fronto-temporal channels, and 3–20 spatially contiguous electrodes were recruited based on Euclidean distance in sensor space. AR(2) coefficients were parameterized by a damping factor *r* ∈ [0.4, 0.65] and peak frequency *f*_0_ ∈ [35, 80]Hz, yielding coefficients *a*_1_ = 2*r*cos(2π*f*_0_/*f*_*s*_) and $a_{2}=-r^{2}$. Each affected channel received an independently scaled copy of the AR(2) noise, with the scale set to achieve a signal-to-artifact ratio drawn uniformly from [1/25, 1/2] relative to the local signal RMS. Artifact durations ranged from 1 to s.

##### 2.3.1.2 DC drift artifacts

Three slow drift segments were generated using an integrated Ornstein–Uhlenbeck (OU) process, *x*(*t*) = *x*(*t*−1)+*u*(*t*), where *u*(*t*) = φ·*u*(*t*−1)+ε(*t*) with autoregressive coefficient φ ∈ [0.92, 0.995] and ε ~ N (0, 1). Each drift affected 1–25 randomly selected channels for 10–20 s, with a 1 s raised-cosine taper applied at onset and offset. Peak-to-peak amplitude was drawn uniformly from 150–800 μV per channel.

Two channels, EEG 018 and EEG 032, were selected for plotting.

#### 2.3.2 Semi-synthetic real data

**Figure 2:**
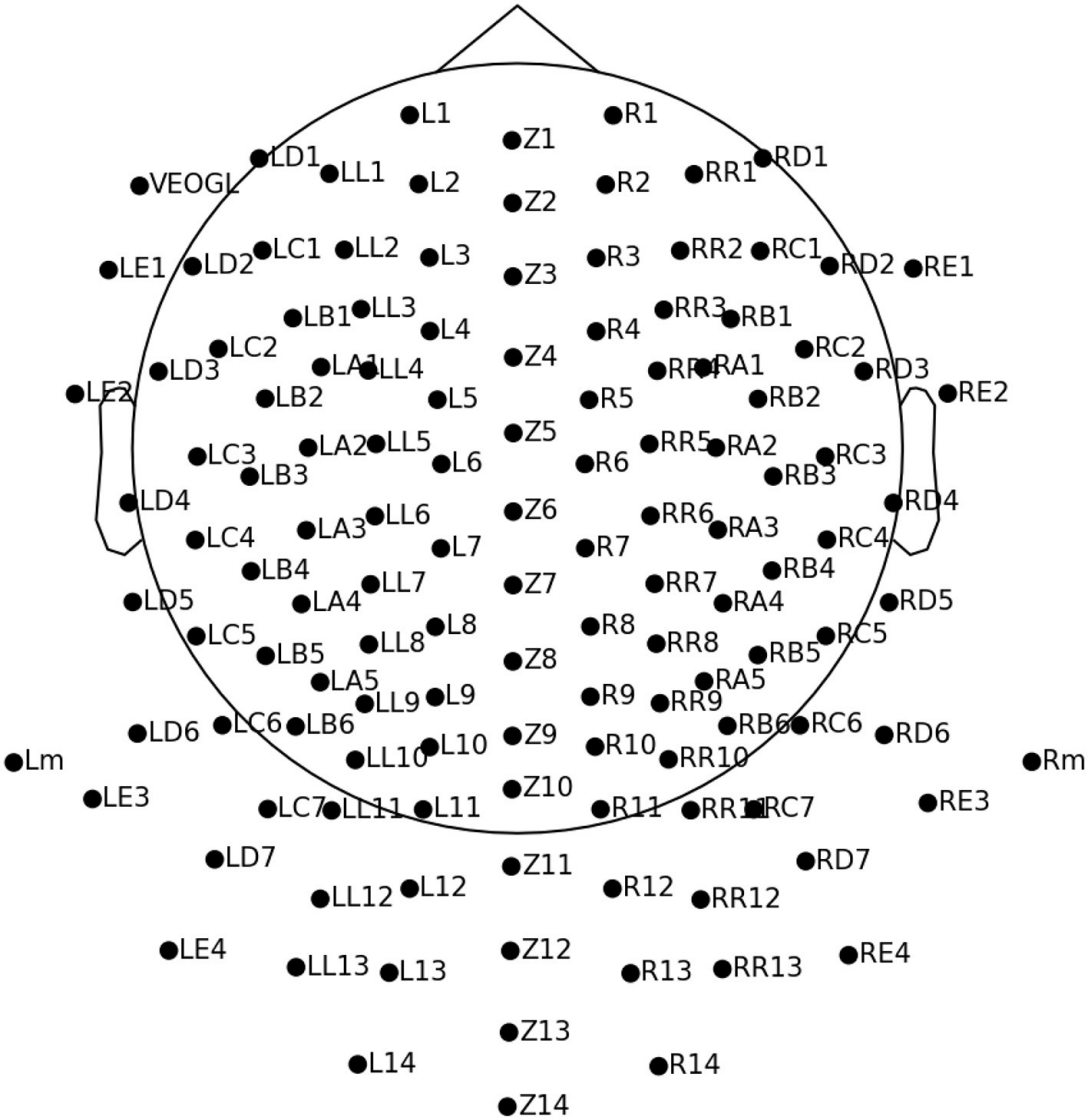
Montage of the 128-channel gel cap for the semi-synthetic real data.

Twenty-nine adult participants (19 female; mean age = 66.5 years) as part of an aging study provided EEG data included in the current analyses of EMG artifact removal. All participants completed experimental sessions consisted of overnight polysomnography recordings at the Massachusetts General Hospital Sleep Center after providing written informed consent. The study protocol was approved by the Massachusetts General Hospital Human Research Committee. All study procedures took place in private sleep lab rooms directed by trained study researchers, with standardized layout of office table and chair during cognitive testing that was typical of in-lab settings of electrophysiological experiments. As the primary aim of the aging study is different from the current proposal of the ICA-S^3^M method, only procedures relevant for the EMG artifact recordings are described below. EEG was recorded using a custom-ordered 128-channel gel cap (Waveguard Duke, ANT Neuro) and the *eego mylab* amplifier system (ANT Neuro), with electrode Z3 serving as the online reference and ground electrode on the left mastoid. Signals were digitized at a sampling rate of 1, 000 Hz. A single vertical electrooculogram (VEOG) referential electrode was placed below the left eye (VEOGL) for monitoring eye movements. Electrode positions are shown in Figure 2.

Participants performed a facial movement paradigm that was intended to elicit strong EMG artifacts. In this task, they mimicked six facial expressions, including brow lowering, brow raising, eye clenching, mouth opening, nose wrinkling, and squinting, presented as short video clips. Each trial consisted of a 5 s rest period, indicated by a red countdown screen, followed by a 4 s video clip of the target facial movement, during which the participant mimicked the expression. An auditory beep signaled the end of each movement period and the transition back to rest. The experiment comprised a practice round (one pass through all six movements) followed by a test phase of 2 blocks × 6 movements = 12 trials. Only the test phase data were analyzed. Digital event codes were sent via a serial port to timestamp events on the EEG recordings. The task paradigm was administered using Presentation software (Neurobehavioral Systems) running on a laptop (Lenovo; Windows 10 Pro 2022). For eight participants, some triggers were missing due to technical errors during the experiment. Missing trigger timing information was recovered based on the known fixed durations of video events (mean = 4.4–4.9 s depending on movement type, std < 0.02 s for 5 of 6 movements) and rest intervals (mean = 5.78 s, std = 0.01 s) measured across all intact recordings.

To enable ground-truth evaluation on real EEG, we constructed a semi-synthetic dataset in which a manually curated clean rest signal was combined with a manually curated EMG artifact signal drawn from the same participant's recording. Construction proceeded in three steps as described below.

##### 2.3.2.1 Clean ground-truth signal

From each trial, the center 4.5 s of the rest period was extracted and all 12 rest segments were stitched end-to-end, yielding a 54 s rest recording per participant. Extended Infomax ICA (variance explained = 0.99) was fitted on this stitched signal. A trained rater reviewed each IC using a four-panel figure displaying its scalp topography, time trace with trial boundaries, 0–50 Hz spectrogram, and power spectrum, and classified the IC as clean or artifactual on a per-trial basis (allowing an IC to be clean in some trials and artifactual in others). The clean ground truth for each trial was reconstructed by back-projecting only the ICs labeled clean for that trial through the mixing matrix.

##### 2.3.2.2 Artifact signal

From each trial, the center 2.0 s of the EMG period was extracted and the 12 EMG segments were stitched end-to-end. Extended Infomax ICA was fitted on the stitched EMG signal, and the same rater classified each IC per trial using the same four-panel figure. Per-trial artifact signals were reconstructed by back-projecting only the ICs labeled artifactual through the mixing matrix.

##### 2.3.2.3 Synthetic noisy signal

For each of the 12 trials, the 2.0 s artifact segment was added to the 4.5 s clean ground-truth segment at a random onset drawn uniformly from [0, 2]*s*, ensuring the artifact fell fully within the rest window. A fixed random seed (= 42) was used for reproducibility. The resulting 54 s synthetic recording per participant contains 12 trials with known clean ground truth, known artifact waveform, and known artifact onset and offset. Across all 29 participants, the rater made 11,025 IC–trial classifications across 1,734 unique ICs (6,777 for EMG segments and 4,248 for rest segments).

##### 2.3.2.4 Rater consistency

A second human rater was not available, so we could not report inter-rater agreement directly on the manual IC–trial labels above. To gauge how reproducible the rater's decisions are, we trained two L_2_-regularized logistic-regression classifiers, one for the EMG period and one for the rest period, to predict the rater's binary labels from 17 objective IC features spanning spectral content (log band powers in δ, θ, α, β, low-γ, high-γ; a broadband HF ratio; the 20–100 Hz 1/*f* slope; an α-peak prominence index), topography (focality, outer-ring edge ratio, frontal–occipital asymmetry, left–right asymmetry, count of active sensors), and time-domain transients (log variance, kurtosis, peak-to-MAD ratio). Models were evaluated under leave-one-subject-out cross-validation across the 29 participants (class_weight = 'balanced'; features *z*-scored within each training fold). The pooled out-of-sample Cohen's κ was 0.61 for the rest period (clean vs. not-clean; *N* = 21, 321 IC × trial; ROC-AUC = 0.93) and 0.60 for the EMG period (clear-artifact vs. not; *N* = 16, 528; ROC-AUC = 0.88), corresponding to substantial agreement (Landis and Koch, 1977). Because the classifier never sees the held-out subject during training, these values are a lower bound on the rater's internal consistency across the cohort. The full analysis, including per-subject performance, standardized feature weights, confusion matrices, and the two interpretive caveats (asymmetric label policy; rater-rule reproducibility is correlated with, but not identical to, intra-rater test–retest, is provided in Appendix 4.2.

For evaluation of ICA-S^3^M, metrics were computed within each of three scalp regions: frontal (15 electrodes: Z1, Z2, Z4, L1, L2, L3, LL1, LL2, R1, R2, R3, RR1, RR2, LD1, and RD1), temporal (20 electrodes: LD3–LD6, RD3–RD6, LC3–LC5, RC3–RC5, LB2–LB4, and RB2–RB4), and occipital (14 electrodes: Z11–Z14, L12–L14, LL12, LL13, R12–R14, RR12, and RR13). Channels marked as bad during preprocessing were excluded on a per-subject basis. For visualization of artifact removal performance, three channels (L2, RB2, and Z11) were selected to represent the frontal, occipital, and temporal regions, respectively.

#### 2.3.3 Naturalistic environment

The naturalistic data were obtained from a single-subject, 10-min EEG recording session collected as part of a larger investigation into brain dynamics during musical improvisation (Principal Investigator: Patrick L. Purdon, Ph.D.; Protocol: “Characterizing brain dynamics during musical improvisation;” Partners Human Research Committee IRB approved). The subject analyzed in this study was a healthy adult with musical improvisation experience, meeting all inclusion criteria specified in the parent protocol. The subject had no history of neurological disease, hearing impairment, or substance abuse, and had no contraindications to EEG recording. The recording session corresponded to Experiment 1 from the main study on music improvisation, in which three participants alternated between playing written music and improvised musical performance. EEG data were acquired using a 65-channel gel cap (Waveguard Duke, ANT Neuro) and the *eego mylab* amplifier system (ANT Neuro) with electrodes positioned according to the extended 10–20 international system. Signals were recorded continuously at 1,000 Hz sampling rate. For evaluation, we used channel 2Z, 8Z, and 4LC representing the frontal, occipital and temporal regions, respectively.

### 2.4 Preprocessing

The following preprocessing steps were then applied using MNE-Python (Gramfort et al., 2013):

1. **Filtering**. Data were bandpass filtered between 1 and 100 Hz using an infinite impulse response (IIR) filter, and notch filter at 60 Hz.
2. **Bad channel detection (impedance)**. Channels with electrode impedance exceeding 200 kΩ were marked as bad. Files in which more than 35% of channels exceeded this threshold were excluded from further analysis.
3. **Bad channel detection (neighbor correlation)**. Neighbor correlation was computed for each channel using the eelbrain library (Brodbeck et al., 2023). Channels whose neighbor correlation fell below the mean minus two standard deviations across all good channels were additionally marked as bad.
4. **Eye blink removal (ICA + ICLabel)**. Extended Infomax ICA was fitted on the data (variance explained threshold of 99.9%, decimation factor of 20). Components classified as eye blinks with probability ≥0.8 by ICLabel (Pion-Tonachini et al., 2019) were identified and removed from the data.
5. **Resampling**. Data were downsampled from 1, 000 Hz to 200 Hz.
6. **Re-referencing**. Data were re-referenced to the common average.
7. **Amplitude-based annotation**. Amplitude-based artifact annotation was performed with a peak threshold of 150 μV and a minimum duration of 10 ms. Segments exceeding the threshold in more than 5% of channels were marked as bad.

The preprocessed recordings were saved in MNE .fif format and served as input to the ICA-S^3^M artifact removal pipeline.

### 2.5 Performance evaluation

#### 2.5.1 Metrics

We evaluated artifact removal performance using two complementary sets of metrics. Ground-truth metrics, consistent with established benchmarks in the EEG denoising literature (Zhang et al., 2021; Raj et al., 2025; Kalita et al., 2024), were applied to the simulated data and to the semi-synthetic real data, where the clean reference signal was available by construction. Blind metrics were applied to the naturalistic recording, for which no ground truth exists.

##### 2.5.1.1 Ground truth metrics

For simulated data, where the clean signal *x*_clean_ is known, we computed the following metrics on each channel:

Relative root-mean-square error (RRMSE). The normalized reconstruction error between the cleaned signal $\hat{x}$ and the ground-truth clean signal:

$$\begin{array}{l} \text{RRMSE}=\frac{||\hat{x}-x_{\text{clean}}{||}_{2}}{||x_{\text{clean}}{||}_{2}} \end{array}$$ (3)

Lower values indicate better reconstruction fidelity.
SNR improvement (ΔSNR). The change in signal-to-noise ratio after artifact removal, expressed in decibels:

$$\begin{array}{l} \text{Δ}\text{SNR}={\text{SNR}}_{\text{after}}-{\text{SNR}}_{\text{before}} \end{array}$$ (4)

where $\text{SNR}=10\log_{10}\left(||x_{\text{clean}}{||}_{2}^{2}/||x-x_{\text{clean}}{||}_{2}^{2}\right)$ and *x* denotes either the corrupted or cleaned signal. Positive values indicate improved signal quality.

Pearson correlation (*r*). The Pearson correlation coefficient between the cleaned signal $\hat{x}$ and the ground-truth clean signal *x*_clean_, providing a scale-invariant measure of waveform similarity.
Band-averaged magnitude-squared coherence. To assess how faithfully each cleaned signal tracks the ground-truth *waveform* within a frequency band (rather than only its integrated power), we computed magnitude-squared coherence between $\hat{x}$ and *x*_clean_ using Welch cross-spectra (nperseg = 256 samples, 50% overlap), and averaged within four canonical bands: delta (1–4 Hz), theta (4–8 Hz), alpha (8–13 Hz), and beta (13–30 Hz). We chose band-averaged coherence in preference to a per-band log-spectral-distance (LSD) metric because LSD compares integrated power within a band independently of waveform alignment, which rewards a failure mode in which a method matches the ground-truth power level in a band without reproducing its time course; coherence, being a cross-spectrum ratio, penalizes such magnitude-only matches because it requires phase agreement as well. Values lie in [0, 1]; higher coherence indicates the cleaned signal more faithfully tracks the ground-truth waveform within the band.

##### 2.5.1.2 Blind metrics

For recordings without ground truth, we used **Spectral Power Ratio (SPR)**, which is the ratio of high-frequency power (30–100 Hz) to low-frequency power (1–30 Hz), computed from multitaper spectral estimates; lower SPR indicates reduced residual EMG contamination.

### 2.6 Artifact model probability at electrode

To validate that ICA-S^3^M correctly localizes EMG contamination in time, we computed a topography-weighted mean artifact probability for each participant and electrode on the semi-synthetic lab dataset, where the noise injection interval of each trial is known by construction. For each kept IC and each 10 s processing window, we extracted the artifact probability time series *p*_art_(*t*) estimated using the sweep-2 state-space model. Windows returning no valid oscillatory model after sweep-1 dOsc were excluded since *p*_art_ is 1 in that case. Within each trial, samples falling inside the recorded artifact injection interval [*t*_inj_, , *t*_inj_+Δ*t*_inj_] were labeled artifact-injected, and all remaining within-trial samples were labeled rest-only, giving a strictly within-trial contrast that is immune to baseline differences between trials. For each IC, all artifact-injected samples and all rest-only samples were pooled separately across windows and trials, and a single mean artifact probability was computed for each condition.

To project IC-level probabilities onto electrode space, we weighted each IC's mean probability by the absolute value of its entry in the ICA mixing matrix for that electrode, and computed the normalized weighted average across all kept ICs:


$$\bar{p}{\text{inject}}^{\left(e\right)}=\frac{\sum_{k}|Aek|,\bar{p}{\text{inject}}^{\left(k\right)}}{\sum_{k}|Aek|},\;\bar{p}{\text{rest}}^{\left(e\right)}=\frac{\sum_{k}|Aek|,\bar{p}{\text{rest}}^{\left(k\right)}}{\sum_{k}|Aek|}$$

where *A*_*ek*_ is the mixing matrix weight for electrode *e* and IC *k*, and the sum is taken over kept ICs for which both conditions contained valid samples. Per-participant bad channels were excluded prior to aggregation. These weighted average probabilities computed separately for the frontal, temporal, and occipital montages, and the resulting (participant × electrode) observations within each region were compared between the artifact-injected and rest-only conditions using a one-sided Wilcoxon signed-rank test (*H*_1_: $\bar{p}{\text{inject}}^{\left(e\right)}>\bar{p}{\text{rest}}^{\left(e\right)}$), with Cohen's *d* reported to quantify effect size.

### 2.7 Complex CNN

To benchmark ICA-S^3^M against a supervised deep learning approach, we replicated the Complex CNN (referred to as CNN for the remaining of the paper) from the EEGdenoiseNet benchmark (Zhang et al., 2021), identified as the best-performing architecture for EMG artifact removal in that study. We also replicated the Simple CNN as a lighter baseline. Our reimplementation used PyTorch rather than the original TensorFlow, following the published architecture and training hyperparameters (Adam optimizer, learning rate 5 × 10^−5^, β_1_ = 0.5, β_2_ = 0.9, MSE loss, batch size 40, 50 epochs).

We validated our reimplementation on the EEGdenoiseNet benchmark dataset (4,514 clean EEG and 5,598 EMG epochs, 512 samples at 256 Hz, mixed at random SNR levels) using the original 80/10/10 train/validation/test split across 10 independent runs. Our CNN achieved RRMSE_*t*_ = 0.673 ± 0.003, closely matching the published result and confirming a faithful replication. One correction to the original training procedure was the model-saving strategy: CNN overfits rapidly on EMG data, with validation loss increasing after approximately 3 epochs. We therefore saved the best model by validation loss from any epoch, rather than the original heuristic of saving after 80% of training epochs.

For evaluation against ICA-S^3^M, we retrained CNN on the full EEGdenoiseNet dataset to maximize the model's exposure to artifact patterns. The full model was applied to each 256 Hz preprocessed recording by segmenting each channel into non-overlapping 2 s windows (512 samples), normalizing each segment by its standard deviation, passing it through the network, de-standardizing the output, and concatenating segments back into a continuous signal. The denoised signals were then resampled to 200 Hz to match the ICA-S^3^M output.

The full-data retrained model was used to generate time-domain traces, power spectral density plots, and representative metric visualizations. For statistical testing, we used the 10 replication models to obtain a distribution of CNN-derived metrics for each trial, and the trial-averaged CNN metric was paired with the corresponding ICA-S^3^M metric as the input to the mixed- effects model described in Section 2.8.

### 2.8 Statistical analysis

For the simulated data and naturalistic recordings, paired Wilcoxon signed-rank tests were applied across all channels, treating each channel as a paired observation (ICA-S^3^M metric value vs. CNN metric value on the same channel). Two-sided tests were used for band-average coherence, RRMSE, SNR improvement, and Pearson correlation. For the simulated data, the unit of observation is the channel, so the simulation generates independent draws under randomized source/artifact configurations. The naturalistic recording is a single session, so within-subject dependence would not apply and the channel-paired Wilcoxon is the appropriate test.

To compare ICA-S^3^M and CNN on the semi-synthetic real data, we treated each (participant × trial) pair as one observation and fit a linear mixed-effects model to the per-trial paired differences for every (region, period, and metric) combination. For each trial, each scalar metric was computed on the trial's artifact-injected span and on its rest-only span, averaged across valid electrodes within a scalp region (frontal, temporal, occipital), and the paired difference $d_{ij}=m_{ij}^{{\text{ICA-S}}^{\text{3}}\text{M}}-m_{ij}^{\text{CNN}}$ was formed for subject *i* and trial *j* (up to 29 × 12 = 348 pairs per combination; 338 in practice after excluding trials with insufficient data). We then fit


$$\begin{array}{l} d_{ij}=\mu +u_{i}+\varepsilon_{ij},\;u_{i}\sim N\left(0,\sigma_{\text{subj}}^{2}\right),\;\varepsilon_{ij}\sim N\left(0,\sigma_{\text{resid}}^{2}\right), \end{array}$$ (5)

by restricted maximum likelihood (statsmodels MixedLM), where *u*_*i*_ is a per-subject random intercept that absorbs within-subject correlation across the 12 trials, and μ is the mean within-subject paired gap; the test of μ = 0 is a two-sided Wald test on the fixed intercept. Seven metrics (RRMSE, SNR improvement, Pearson correlation, and band-averaged magnitude-squared coherence in delta (1–4 Hz), theta (4–8 Hz), alpha (8–13 Hz), and beta (13–30 Hz)) for two periods (artifact-injected, clean) and three regions were tested, giving a Bonferroni family of 7 × 2 × 3 = 42 tests and a family-wise significance threshold of α = 0.05/42 ≈ 1.19 × 10^−3^. Subject-specific bad channels were excluded prior to within-region averaging. For each combination we report the mean paired difference μ and its 95% confidence interval, and *p*-value.

## 3 Results

### 3.1 Simulation

We evaluated ICA-S^3^M and CNN on simulated 59-channel EEG data contaminated with AR(2) EMG artifacts (signal-to-artifact ratio range 1/25 to 1/2; duration 1–5 s) and integrated Ornstein–Uhlenbeck DC drift. Performance was assessed using paired Wilcoxon signed-rank tests across all 59 channels.

#### 3.1.1 Signal fidelity

**Figure 3:**
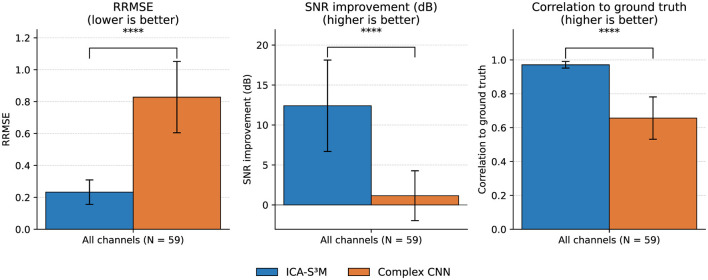
For simulation data, time-domain reconstruction performance during artifact periods. Bar height shows the mean and error bars show ±1 SD across 59 electrodes for ICA-S^3^M (blue) and CNN (orange). Lower RRMSE, higher SNR improvement, and higher correlation indicate better reconstruction of the clean ground-truth signal. ****p < 0.0001.

ICA-S^3^M substantially outperformed CNN on all ground-truth metrics. ICA-S^3^M achieved an RRMSE of 0.23 ± 0.08 compared to 0.83 ± 0.22 for CNN (*W* = 0, *p* = 2.39 × 10^−11^). SNR improvement was 12.4 ± 5.7 dB for ICA-S^3^M versus 1.2 ± 3.1 dB for CNN (*W* = 0, *p* = 2.39 × 10^−11^). Pearson correlation with the clean ground truth was 0.971 ± 0.020 for ICA-S^3^M and 0.657 ± 0.125 for CNN (*W* = 0, *p* = 2.39 × 10^−11^), indicating that ICA-S^3^M reconstructions preserved nearly all of the original waveform structure, whereas CNN introduced distortion. These results are illustrated in Figure 3.

#### 3.1.2 Spectral preservation

Overall band-averaged magnitude-squared coherence between cleaned and ground-truth signals was substantially higher for ICA-S^3^M than for CNN in four of five bands, with significant paired Wilcoxon signed-rank tests across all 59 channels (Bonferroni-corrected α = 0.05/5 = 0.01). ICA-S^3^M achieved significantly higher coherence in the delta (0.86 ± 0.13 vs. 0.39 ± 0.10, *p* = 3.1 × 10^−11^, Cohen's *d* = +2.78), theta (0.85 ± 0.16 vs. 0.69 ± 0.16, *p* = 5.0 × 10^−7^, *d* = +0.73), beta (0.85 ± 0.14 vs. 0.60 ± 0.16, *p* = 6.6 × 10^−10^, *d* = +1.20), and gamma (0.79 ± 0.13 vs. 0.15 ± 0.03, *p* = 2.5 × 10^−11^, *d* = +4.45) bands. In the alpha band, ICA-S^3^M had higher coherence on average (0.84 ± 0.16 vs. 0.80 ± 0.18, *d* = +0.21) but the difference did not reach Bonferroni-corrected significance (*p* = 9.8 × 10^−2^).

#### 3.1.3 Visual inspection

**Figure 4:**
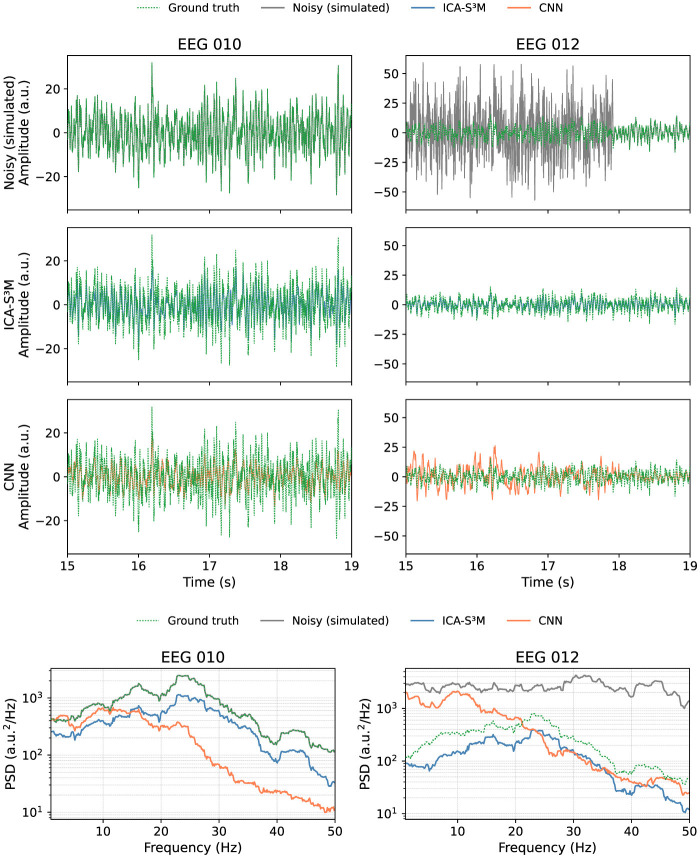
Simulation results for a representative 3 s segment. **Top:** Time-domain traces showing the ground truth (GT), the artifact-contaminated signal (Noisy), and the reconstructions produced by ICA-S^3^M and CNN. **Bottom:** Power spectral densities for two example electrodes. At EEG 018 (left), the ground truth contains a narrow low-beta peak and a small gamma peak, with no EMG noise; ICA-S^3^M recovers both, whereas CNN misrepresents the low-beta peak as a broad alpha-band bump. At EEG 032 (right), which has substantial EMG artifacts, CNN “hallucinates” an alpha peak that is absent from the ground truth, erroneously inferring it from the broadband artifact spectrum.

Visual inspection of the time-domain traces (Figure 4 top) reveals that ICA-S^3^M recovers the oscillatory structure and phase of the simulated EEG signal better than CNN, as illustrated in channels EEG 018 and EEG 032. EEG 018 exemplifies a channel that is relatively clean, free of EMG artifacts, whereas EEG 032 is representative of a channel with significant EMG artifacts. From the power spectral density plots (Figure 4 bottom), in EEG 018 we see that CNN substantially attenuates the amplitudes of alpha, beta, and gamma oscillations that are present in the signal. Meanwhile, in EEG 032, CNN “hallucinates” a spurious alpha peak that exceeds the amplitude of the peak present in the ground truth data (Figure 4 bottom); this hallucinated alpha oscillation is also evident in the time domain trace (Figure 4 third row). ICA-S^3^M, by contrast, more faithfully preserves the spectral profile of the ground truth in both EEG 018 and EEG 032.

### 3.2 Verifying CNN alpha oscillation “hallucination"

To isolate and confirm this apparent “hallucination” effect, we repeated the simulation analysis above in Section 2.3.1, but generated the source-level ground truth with no alpha oscillations. Any alpha-band activity in CNN output must therefore be attributed to the method itself rather than to successful recovery of a genuine oscillation. As shown in Supplementary Figure S2, CNN produces a clear spurious alpha peak and substantially distorts the overall spectral shape in both an artifact-free channel (EEG 010) and an artifact-contaminated channel (EEG 012). ICA-S^3^M, by contrast, preserves the alpha-free ground-truth spectrum at both channels.

### 3.3 Semi-synthetic real data

#### 3.3.1 Signal fidelity

Table 3 summarizes every (metric × region × period) combination with average and standard deviations with its Bonferroni-corrected significance marker.

**Table 3:** Paired per-trial comparison of ICA-S^3^M vs. CNN on the semi-synthetic lab dataset.

	Frontal		Temporal		Occipital	
Metric	Artifact	Clean	Artifact	Clean	Artifact	Clean
RRMSE	+0.039 ± 0.569n.s.	+0.219 ± 0.106^***^	+0.126 ± 0.128^***^	+0.148 ± 0.072^***^	+0.164 ± 0.118^***^	+0.187 ± 0.084^***^
SNR imp. (dB)	+1.37 ± 1.86^***^	—	+1.62 ± 1.21^***^	—	+2.58 ± 1.91^***^	—
Correlation	+0.123 ± 0.082^***^	+0.153 ± 0.058^***^	+0.129 ± 0.065^***^	+0.118 ± 0.045^***^	+0.140 ± 0.063^***^	+0.134 ± 0.046^***^
Coh. δ	+0.121 ± 0.078^***^	+0.076 ± 0.111^***^	+0.147 ± 0.081^***^	+0.077 ± 0.104^***^	+0.175 ± 0.101^***^	+0.079 ± 0.115^***^
Coh. θ	+0.090 ± 0.068^***^	+0.032 ± 0.056^***^	+0.092 ± 0.063^***^	+0.028 ± 0.051^***^	+0.099 ± 0.074^***^	+0.033 ± 0.062^***^
Coh. α	+0.073 ± 0.067^***^	−0.006 ± 0.039n.s.	+0.053 ± 0.059^***^	−0.002 ± 0.031n.s.	+0.040 ± 0.068^***^	+0.004 ± 0.033n.s.
Coh. β	+0.106 ± 0.063^***^	+0.042 ± 0.064^***^	+0.113 ± 0.055^***^	+0.037 ± 0.059^***^	+0.131 ± 0.065^***^	+0.049 ± 0.073^***^

Each entry reports the mean ± standard deviation of the per-trial paired difference (ICA-S^3^M − CNN) across 338 (participant × trial) observations per region, sign-normalized so that positive values favor ICA-S^3^M. Significance markers come from the linear mixed-effects model described in Section 2.8.

^***^ICA-S^3^M significantly better than the CNN at the Bonferroni family-wise threshold α = 0.05/42 ≈ 1.19 × 10^−3^ (*p* < α, two-sided test of the model intercept). “n.s.” indicates no significant difference at the same threshold. SNR improvement is undefined for clean periods (residual error approaches zero) and is omitted. Sign convention: positive values favor ICA-S^3^M in all metrics (RRMSE differences are sign-flipped).

##### 3.3.1.1 Artifact-injected periods

ICA-S^3^M outperformed the CNN on every time-domain fidelity metric in every region (all *p* < 10^−8^), with one exception: at frontal electrodes, the RRMSE gap between methods was small in absolute terms (0.997 ± 0.830 for ICA-S^3^M vs. 1.036 ± 0.617 for the CNN; mean paired difference +0.039 ± 0.569, *p* = 0.47) and not statistically distinguishable from zero under the mixed-effects model. This likely reflects the extreme amplitude of the injected facial EMG at sites sitting closest to the muscles driving it. However, the performance advantage of ICA-S^3^M was readily apparent in other regions. For correlation with the ground truth, the mean paired advantage of ICA-S^3^M over CNN went from +0.123 ± 0.082 frontally to +0.129 ± 0.065 temporally and +0.140 ± 0.063 occipitally. In temporal electrodes, RRMSE dropped from the noisy baseline to 0.717 ± 0.190 for ICA-S^3^M vs. 0.843 ± 0.171 for the CNN (mean paired difference +0.126 ± 0.128, *p* < 10^−16^); in occipital electrodes, RRMSE reached 0.598 ± 0.202 vs. 0.762 ± 0.135 (mean paired difference +0.164 ± 0.118, *p* < 10^−16^).

**Figure 5:**
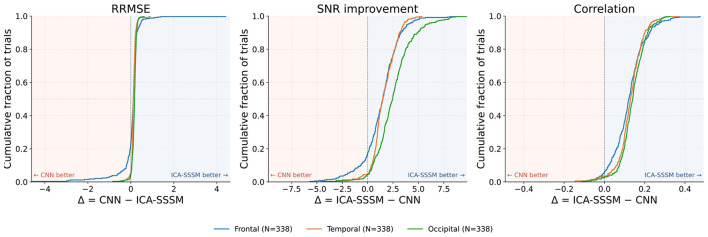
Semi-synthetic data, time-domain reconstruction performance during artifact-injected periods. Each panel (RRMSE, SNR improvement, correlation with the clean ground truth) shows the ECDF of the paired per-trial Δ across (participant × trial) observations, one line per region (frontal, temporal, occipital; *N* = 338 each, 1, 014 per panel). Δ is sign-normalized so that positive always favors ICA-S^3^M (Δ= CNN − ICA-S^3^M for RRMSE, which is lower-is-better; Δ= ICA-S^3^M − CNN for SNR improvement and correlation). Values right of zero (blue) favor ICA-S^3^M; left (coral) favor the CNN. All three metrics tilt decisively right. ICA-S^3^M is better by >0.1 on 56%–81% of RRMSE trials, 82%–95% of SNR-improvement trials, and 63%–79% of correlation trials, confirming that ICA-S^3^M removes the injected EMG more effectively than CNN across the scalp. The only non-trivial left tail is at frontal electrodes (15% of RRMSE and 15% of SNR-improvement trials with ICA-S^3^M worse by >0.1), where the high-amplitude facial EMG occasionally drives ICA-S^3^M's decomposition to overshoot the true signal; the ICA-better tail still dominates in both cases.

SNR improvement showed the same regional pattern. ICA-S^3^M achieved +5.36 ± 4.90 dB frontally, +2.18 ± 5.28 dB temporally, and −0.48 ± 5.91 dB occipitally, vs. +3.99 ± 4.85 dB, +0.55 ± 5.25 dB, and −3.05 ± 6.30 dB for the CNN. At occipital sites both methods have a *negative* mean improvement, meaning that any residual distortion introduced by cleaning exceeds the noise it removes because the EMG is small in occipital sites. However, ICA-S^3^M's distortion is consistently ≈2.6 dB smaller than the CNN's (mean paired difference +2.58 ± 1.91 dB, *p* < 10^−16^). Temporal correlation exceeded the CNN's in every region (0.625 ± 0.169 vs. 0.502 ± 0.152 frontally; 0.687 ± 0.145 vs. 0.557 ± 0.140 temporally; 0.777 ± 0.141 vs. 0.637 ± 0.143 occipitally). These metrics are visualized in Figure 5.

##### 3.3.1.2 Clean periods

**Figure 6:**
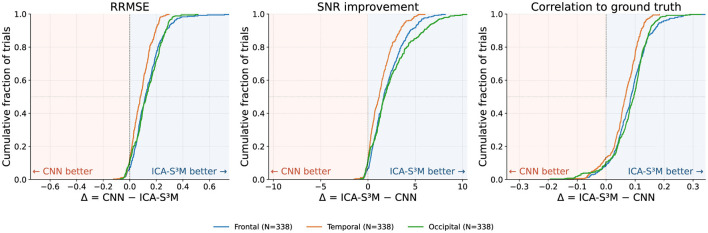
Semi-synthetic data, time-domain reconstruction performance during clean (rest-only) periods. Each panel (RRMSE, SNR improvement, correlation with the clean ground truth) shows the ECDF of the paired per-trial Δ across (participant × trial) observations, one line per region (frontal, temporal, occipital; *N* = 338 each, 1, 014 per panel). Δ is sign-normalized so that positive always favors ICA-S^3^M (Δ= CNN − ICA-S^3^M for RRMSE, which is lower-is-better; Δ= ICA-S^3^M − CNN for SNR improvement and correlation). Values right of zero (blue) favor ICA-S^3^M; left (coral) favor the CNN. RRMSE and correlation tilt decisively right. ICA-S^3^M is better by >0.1 on 75%–92% of RRMSE trials and 75%–86% of correlation trials, against < 1% in the opposite direction in every region, confirming that ICA-S^3^M leaves the clean signal closer to the ground truth than the CNN does. SNR improvement is shown only for completeness; on clean periods the residual error approaches zero and the dB ratio is dominated by floating-point behavior, so the panel is not a meaningful comparison.

Outside the artifact injection windows the ground truth contains no EMG, so SNR improvement is dominated by floating-point behavior when the residual error approaches zero; we therefore focus on RRMSE and correlation. Here ICA-S^3^M left the clean signal essentially untouched while the CNN introduced systematic distortion. RRMSE was substantially lower for ICA-S^3^M in all regions (frontal 0.517 ± 0.138 vs. 0.736 ± 0.121; temporal 0.581 ± 0.152 vs. 0.729 ± 0.102; occipital 0.521 ± 0.162 vs. 0.708 ± 0.101), with mean paired differences of +0.219, +0.148, and +0.187 respectively and all *p* < 10^−16^. Temporal correlation with the ground truth was likewise higher (0.847 ± 0.081 vs. 0.694 ± 0.091 frontally; 0.801 ± 0.103 vs. 0.684 ± 0.100 temporally; 0.841 ± 0.090 vs. 0.707 ± 0.096 occipitally), with mean paired differences of +0.153, +0.118, and +0.134 and all *p* < 10^−16^. In short, ICA-S^3^M does not alter the underlying neural signal when no artifact is present, whereas the CNN does. These results are visualized in Figure 6.

#### 3.3.2 Spectral fidelity

**Figure 7:**
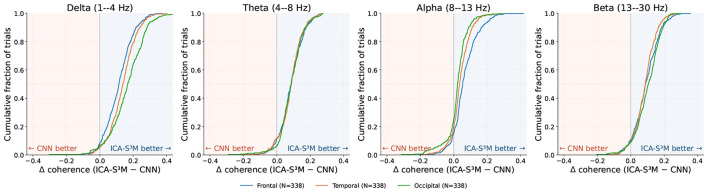
Semi-synthetic data, band-averaged magnitude-squared coherence between cleaned and clean ground-truth signals during artifact-injected periods. Each panel (delta 1–4 Hz, theta 4–8 Hz, alpha 8–13 Hz, beta 13–30 Hz) shows the ECDF of Δ= coherence$\text{ICA}-S^{3}M^{-}$ coherence_CNN_ across paired (participant × trial) observations, one line per region (frontal, temporal, occipital; *N* = 338 each, 1, 014 per panel). Values right of zero (blue) favor ICA-S^3^M; left (coral) favor the CNN. Unlike the clean-period counterpart, few trials are tied (|Δ| ≤ 0.02 on only 3%–26% across cells) because the injected EMG forces both methods to actively modify the signal. Delta, theta, and beta tilt decisively right: ICA-S^3^M better by >0.1 on 62%–80% of delta trials, 41%–47% of theta, and 50%–70% of beta, with < 1% in the opposite direction in each band. Alpha's advantage is smaller and shrinks posteriorly: ICA-S^3^M better by >0.1 on 27% of frontal, 18% of temporal, and 13% of occipital trials.

Band-averaged magnitude-squared coherence quantifies how well each cleaned signal tracks the ground-truth *waveform* within a frequency band, not just its integrated power. During artifact periods, ICA-S^3^M achieved higher coherence than the CNN in every band and every region. All twelve region × band cells were Bonferroni-significant, with *p* < 10^−16^ in eleven of them and *p* = 5.0 × 10^−7^ in the remaining occipital alpha cell. Mean paired advantages were largest in delta, theta, and beta (mean paired differences between +0.090 and +0.175 across regions and bands). Critically, ICA-S^3^M also outperformed the CNN in the alpha band across all three regions (frontal +0.073 ± 0.067, temporal +0.053 ± 0.059, occipital +0.040 ± 0.068; Figure 7).

**Figure 8:**
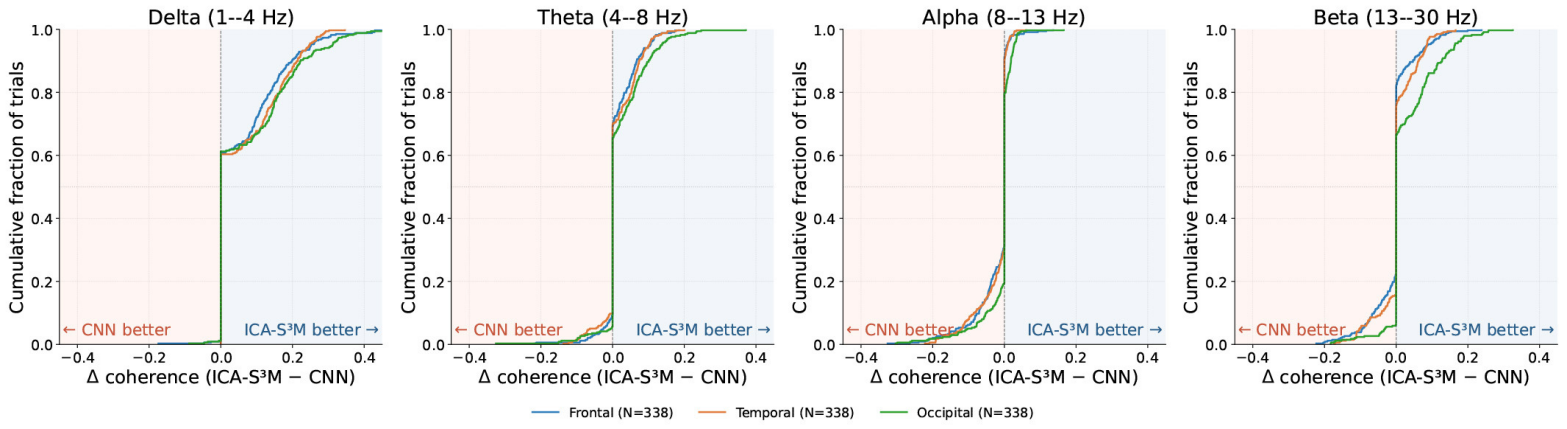
Semi-synthetic data, band-averaged magnitude-squared coherence between cleaned and clean ground-truth signals during clean (rest-only) periods. Each panel (delta 1–4 Hz, theta 4–8 Hz, alpha 8–13 Hz, beta 13–30 Hz) shows the ECDF of Δ= coherence$\text{ICA}-S^{3}M^{-}$ coherence_CNN_ across paired (participant × trial) observations, one line per region (frontal, temporal, occipital; *N* = 338 each, 1, 014 per panel). Values right of zero (blue) favor ICA-S^3^M; left (coral) favor the CNN. The vertical rise at Δ = 0 is the large fraction of trials (|Δ| ≤ 0.02) on which both methods leave the clean signal essentially untouched (≈60% in delta, theta, and beta; ≈75% in alpha). Delta shows ICA-S^3^M dominant (better by >0.1 on 35%–36% of trials, < 1% in the opposite direction); theta and beta have smaller, mostly ICA-favored tails (theta 12%–15% better, beta 22%–28% better, < 1% worse in either band). Alpha is essentially symmetric in both tails (0%–2% of trials show ICA-S^3^M better by >0.1, 2% show the CNN better) across all three regions, consistent with the mixed-effects model finding no significant clean-alpha difference between methods.

During clean periods, both methods leave most of the rest-only signal essentially untouched, so a large fraction of trials have near-perfect coherence (≥0.99) with the ground truth for both ICA-S^3^M and CNN simultaneously; the LMM contrasts below are driven by the remaining trials where the two methods diverge. Per-trial paired differences $\Delta ={\text{coh}}_{{\text{ICA-S}}^{\text{3}}\text{M}}-{\text{coh}}_{\text{CNN}}$ (mean ± SD across *N* = 338 subject-trial pairs per cell, positive favors ICA-S^3^M) illustrate the spectral-fidelity advantage of ICA-S^3^M in delta, theta, and beta in every region: delta +0.076 ± 0.111, +0.077 ± 0.104, +0.079 ± 0.115 (frontal/temporal/occipital, all *p* < 10^−12^); theta +0.032 ± 0.056, +0.028 ± 0.051, +0.033 ± 0.062 (all *p* < 10^−12^); beta +0.042 ± 0.064, +0.037 ± 0.059, +0.049 ± 0.073 (all *p* < 10^−12^). Clean-period alpha, in contrast, showed no significant difference between methods in any region (frontal +0.006 ± 0.038, *p* = 4.1 × 10^−3^; temporal −0.002 ± 0.031, *p* = 2.3 × 10^−1^; occipital +0.004 ± 0.033, *p* = 4.1 × 10^−2^; none surviving Bonferroni at α = 1.19 × 10^−3^). These results are shown in Figure 8.

#### 3.3.3 Visual inspection

**Figure 9:**
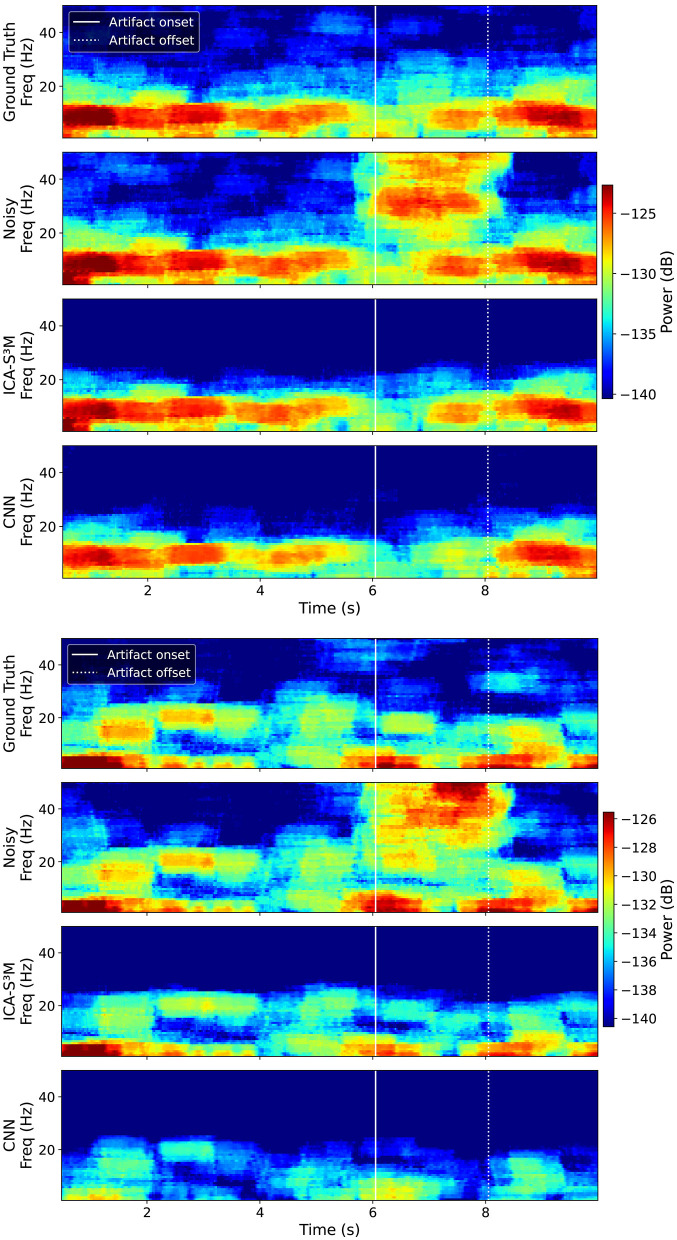
Temporal-region average spectrograms (14 electrodes, one block) from two representative participants in the semi-synthetic dataset. For each participant, rows show **(top to bottom)** the clean ground truth, the artifact-injected noisy signal, the ICA-S^3^M reconstruction, and the CNN reconstruction. Both methods suppress the injected EMG artifact, but the CNN also erroneously removes genuine neural oscillations: alpha activity in participant 1 **(top)** and delta, theta, and beta activity in participant 2 **(bottom)**. ICA-S^3^M preserves these oscillations while removing the artifact.

As illustrated in Figure 9, both ICA-S^3^M and CNN methods preserve alpha activity comparably upon visual inspection. However, the bottom row shows that ICA-S^3^M better preserves beta-band activity, which exhibits a pattern consistent with movement-related synchronization and desynchronization. Across both participants, slow-wave activity is retained in the ICA-S^3^M output but attenuated in the CNN output.

**Figure 10:**
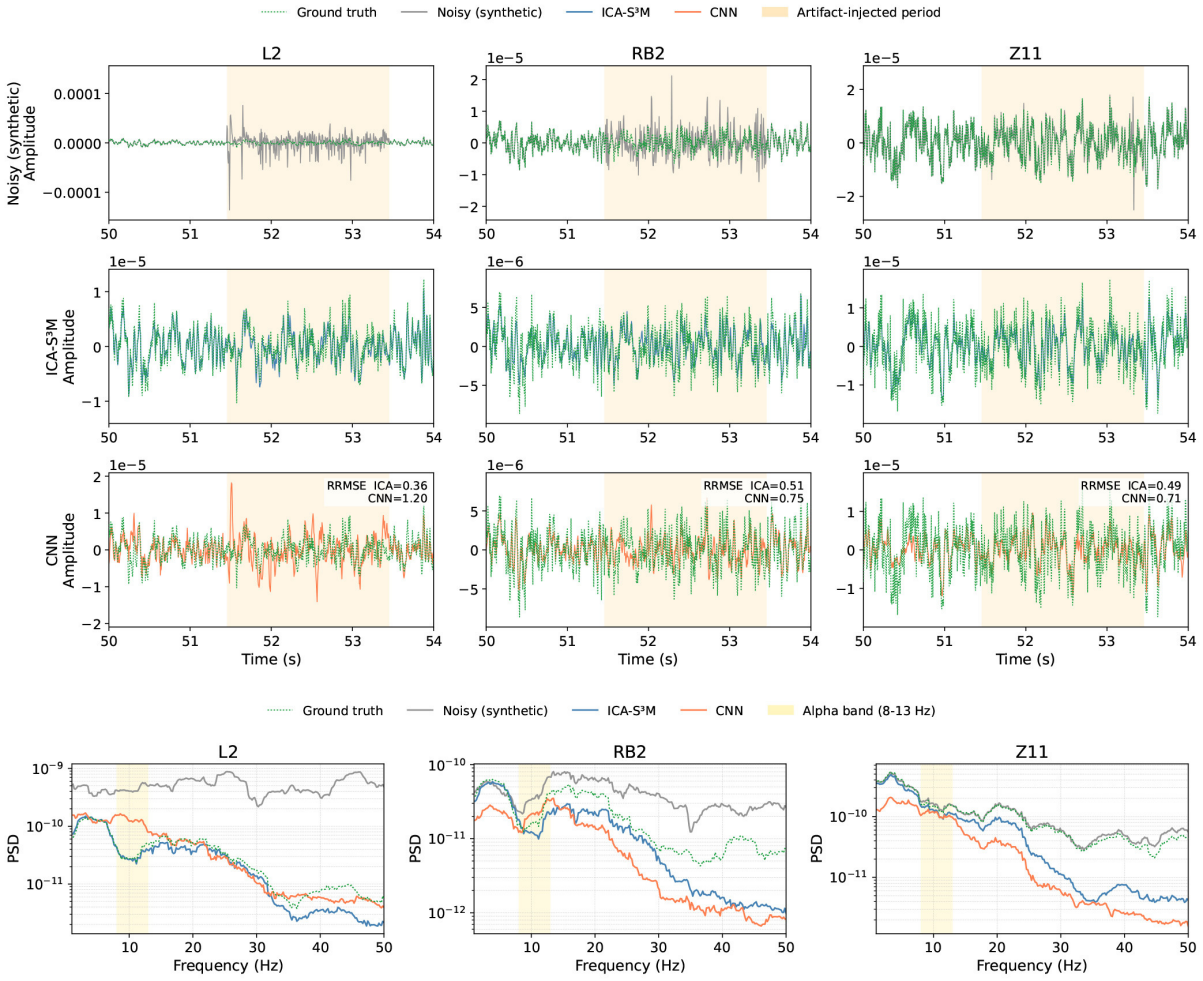
Semi-synthetic results from one participant for representative 4-second segments. **Top**: time-domain traces of the ground truth, contaminated input, and the outputs of ICA-S^3^M and the CNN at L2 (frontal), RB2 (temporal), and Z11 (occipital). **Bottom**: corresponding power spectra. Both methods visually attenuate the high-frequency EMG burst, but the CNN systematically distorts the underlying spectrum: it suppresses the theta oscillation at RB2 and low-beta oscillations at L2 and Z11, and hallucinates spurious alpha oscillations from the artifact at all three electrodes.

As shown in Figure 10, while both methods visibly attenuate the broadband EMG burst, the two methods diverge sharply in how they treat the underlying neural oscillations. ICA-S^3^M leaves the pre- and post-artifact spectral content essentially intact, whereas the CNN introduces systematic, band-specific distortions across all three illustrated electrodes. At electrode RB2, the CNN attenuates the ongoing theta rhythm that is clearly preserved by ICA-S^3^M. At electrodes L2 and Z11, the CNN suppresses low-beta activity that was present before and after the artifact window. Most strikingly, at all three electrodes the CNN injects a spurious narrowband alpha peak precisely where the EMG burst occurred—the same “alpha hallucination” failure mode observed in the simulation study, now reproduced on real scalp recordings where no ground-truth alpha oscillation was present in the surrounding signal.

**Figure 11:**
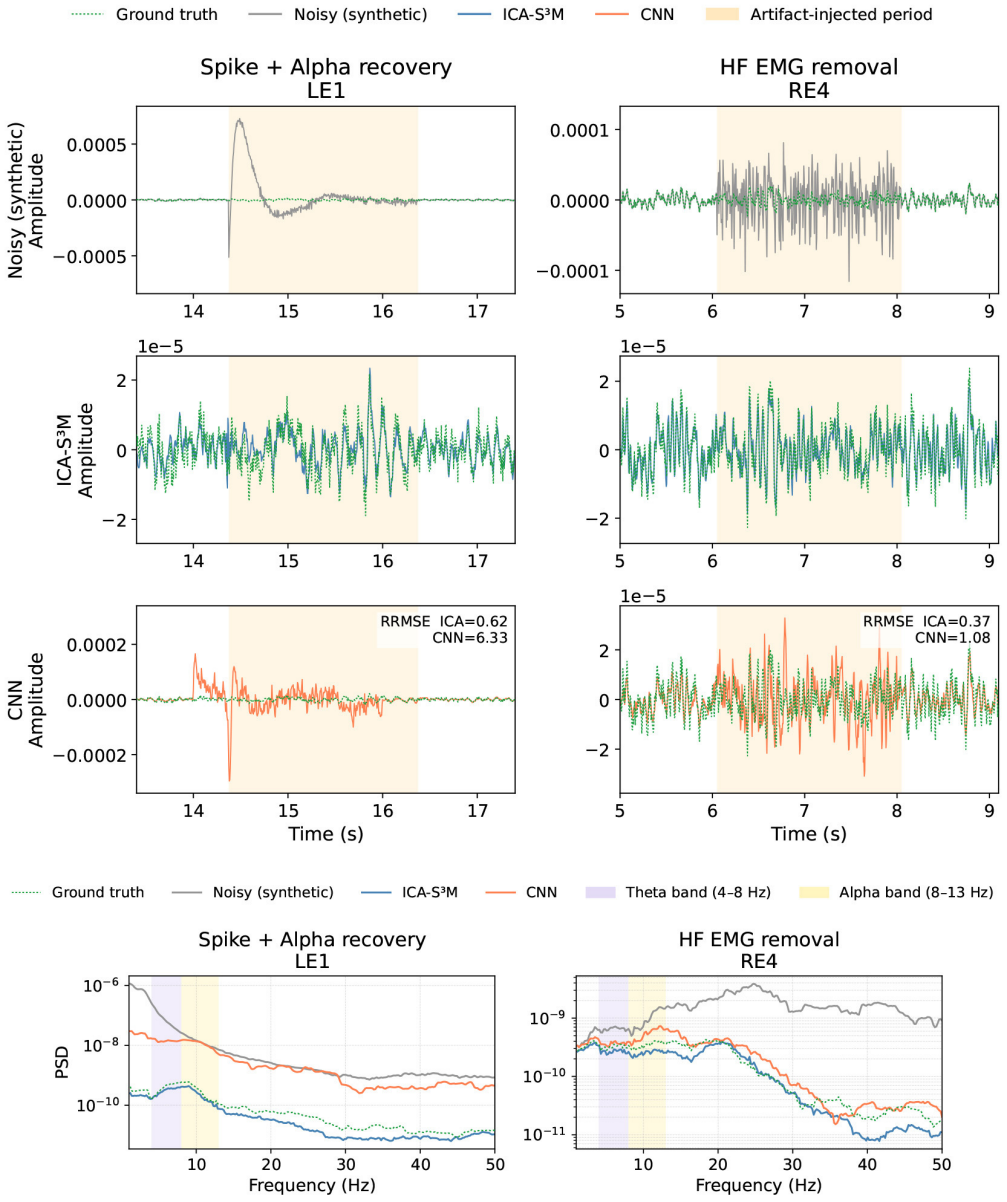
Semi-synthetic results for two representative 4-second segments illustrating ICA-S^3^M artifact removal in the presence of different types of artifacts. **Top**: time-domain traces of the ground-truth signal, the contaminated input, and the outputs of ICA-S^3^M and the CNN. **Bottom**: corresponding power spectral densities. In segment RE1, a large spike artifact dominates the contaminated input; ICA-S^3^M removes it almost entirely, recovering the underlying theta oscillation with near-perfect fidelity, while the CNN removed only portions of the spike artifact. In segment L2, the contaminated signal contains broadband high-frequency EMG; ICA-S^3^M suppresses the EMG while faithfully preserving the ground-truth waveform and spectrum, while the CNN leaves residual high-frequency energy and again hallucinates a spurious alpha oscillation that was absent from ground truth.

Figure 11 presents two representative 4-second segments that further highlight the contrasting behavior of the two methods. In the first segment (RE1), the contaminated input contains a large spike artifact; ICA-S^3^M removes it almost entirely, recovering the underlying alpha oscillation with near-perfect spectral fidelity, whereas the CNN produces a residual deflection of opposite polarity and fabricates a prominent alpha peak that is absent from the ground truth. In the second segment (L2), the contaminated signal contains broadband high-frequency EMG. ICA-S^3^M suppresses the EMG component while faithfully preserving the ground-truth waveform and its spectral content. Meanwhile the CNN leaves behind residual high-frequency energy and again introduces a spurious alpha oscillation, consistent with the hallucination pattern observed across the full simulation dataset.

#### 3.3.4 Alpha-band power inflation (“alpha hallucination”)

To quantify the spectral signature of the failure mode visible in Figures 10, 11, we computed band-integrated alpha power (8–13 Hz, trapezoidal integration of the multitaper PSD; linear units) on the same per-(channel × trial × period) windows used in the per-trial analysis, for four paired signals: clean ground truth (GT), GT plus the injected EMG (“noisy"), ICA-S^3^M, and CNN. For each (region × period × method) cell we fitted a linear mixed-effects model on the log-ratio of power for each method to GT with a per-subject random intercept, log(power_method_/power_GT_)~1+(1|subject), and exponentiated the fixed-effect intercept to report a linear ratio versus GT.

Frontal channels during EMG-injection windows constitute the worst case for spectral contamination of the 8–13 Hz band. There, EMG injection inflated band-integrated alpha power 1.83 × over the clean ground truth (95% CI 1.62–2.07; *p* = 3 × 10^−22^; *n* = 4, 543 channel-trials, 29 subjects). ICA-S^3^M removed essentially all of this inflation, with a residual ratio of 0.70 × GT (95% CI 0.62–0.79; *p* = 7 × 10^−9^), indicating slight over-correction. CNN, by contrast, left a 30% residual inflation (1.30 × GT; 95% CI 1.16–1.45; *p* = 3 × 10^−6^): 28 of 29 individual subjects showed a frontal-artifact CNN/GT ratio above 1, with a per-subject median of 1.81 (Table 4). Because no neural process can generate alpha-band power that exceeds the clean rest baseline during a 2 s muscle-contraction window in the same underlying brain state, this excess is the spectral signature of alpha-band energy that originated in the EMG source and was passed through the CNN as if it were neural alpha.

**Table 4:** Linear-units alpha-band power (8–13 Hz) relative to clean ground truth, frontal channels, EMG-injection windows.

Signal	Mean (*μV*^2^)	Ratio vs. GT (95*%* CI)	*p*
Ground truth (clean rest)	1.12 × 10^−9^	1.00	—
GT + injected EMG	2.84 × 10^−9^	1.83 (1.62–2.07)	3 × 10^−22^
ICA-S^3^M cleaned	1.07 × 10^−9^	0.70 (0.62–0.79)	7 × 10^−9^
CNN cleaned	2.15 × 10^−9^	**1.30** **(1.16**–**1.45)**	3 × 10^−6^

Linear ratio of each method to GT estimated by a linear mixed-effects model on log(method/GT) with a per-subject random intercept, exponentiated; *n* = 4, 543 channel-trials, 29 subjects. The bolded value in the CNN row highlights the alpha ‘hallucination' effect, in which the CNN-cleaned alpha power is substantially higher than the ground truth.

#### 3.3.5 Semi-synthetic real data summary

We tested 42 scenarios spanning 7 performance metrics, 2 artifact conditions, and 3 spatial domains, and found that ICA-S^3^M significantly outperformed the CNN in 38 of those scenarios. In particular, ICA-S^3^M outperformed the CNN in 20 of 21 artifact-period scenarios and in 18 of 21 clean-period scenarios. The remaining four cells were inconclusive under the Bonferroni threshold—frontal RRMSE during artifact periods (*p* = 0.47), and alpha-band coherence during clean periods at frontal, temporal, and occipital sites (*p* = 0.16, 0.35, and 0.17, respectively)—and no cell significantly favored the CNN. Taken together, ICA-S^3^M recovers the underlying signal more effectively than the CNN across multiple spatial domains, whether artifacts are present or not, and across both time- and frequency-domain metrics. Crucially, when artifacts are absent, ICA-S^3^M does not alter the underlying signal, and furthermore does not “hallucinate" EEG activity that is not present.

#### 3.3.6 Artifact probability

On the semi-synthetic lab dataset, the topography-weighted mean artifact probability assigned by ICA-S^3^M was markedly higher during artifact-injected periods than during rest-only periods across all three electrode montages. For the pooled (participant × electrode) comparison, the mean weighted artifact probability during injected vs. rest-only periods was 0.402 ± 0.111 vs. 0.100 ± 0.036 frontally (*N* = 390; paired-difference LMM with subject random intercept: $\hat{\mu }=0.300$, 95% CI [0.258, 0.342], *p* = 2.8 × 10^−44^), 0.371 ± 0.120 vs. 0.116 ± 0.045 temporally (*N* = 573; $\hat{\mu }=0.255$, 95% CI [0.209, 0.301], *p* = 2.2 × 10^−27^), and 0.351 ± 0.135 vs. 0.117 ± 0.049 occipitally (*N* = 403; $\hat{\mu }=0.233$, 95% CI [0.179, 0.288], *p* = 4.1 × 10^−17^). The artifact-injected vs. rest contrast was strictly positive in every (participant × electrode) pair across all three regions, and the separation was largest frontally, consistent with the anterior bias expected from facial-EMG sources. These results confirm that ICA-S^3^M selectively assigns high artifact probability to the known EMG-contaminated samples while suppressing artifact probability on the clean rest samples of the same trials, and that the within-trial separation is robust across the scalp.

**Figure 12:**
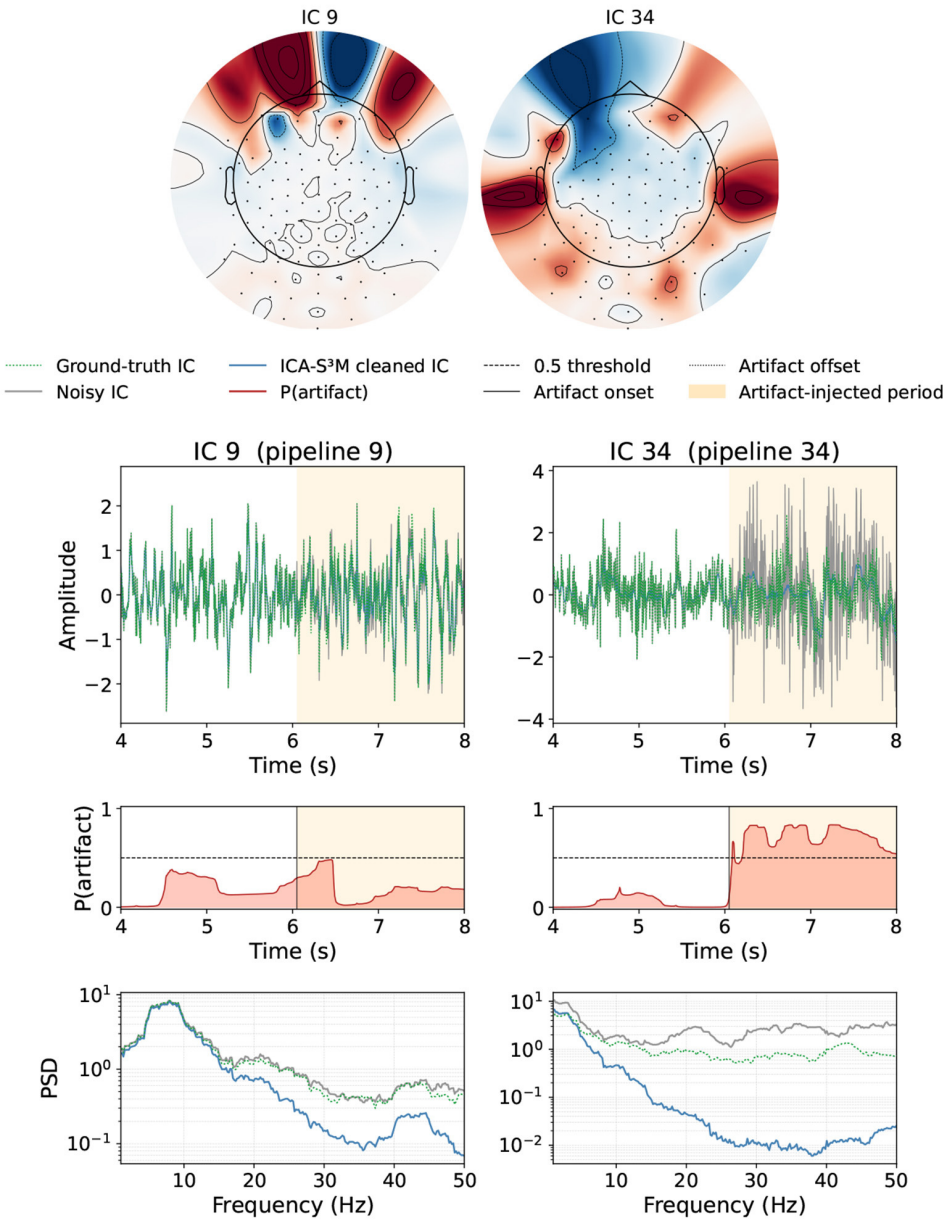
Illustration of the ICA-S^3^M artifact-scrubbing pipeline for one representative participant. **(Top)** Scalp topographies of two independent components (IC09 and IC34) identified by ICA, showing spatial patterns consistent with EMG contamination. **(Bottom)** Detailed view of IC09: (left) time trace of the component alongside the sweep-2 state-space model's frame-wise artifact probability, plotted on a shared time axis; and (right) power spectral density of the ground-truth, noisy, and ICA-S^3^M-cleaned component. The estimated artifact probability closely tracks the visually apparent extent of contamination, demonstrating the interpretability of the state-space model's output.

Figure 12 illustrates the within-IC artifact scrubbing process for two representative ICs from one participant. In both components, the artifact probability estimated by the sweep-2 model closely tracks the visually apparent EMG contamination, rising sharply during broadband bursts and remaining low during quiescent segments. Artifact probability is consistently elevated during the EMG trial period relative to the adjacent rest period. Importantly, this figure reveals that both ICs contain a mixture of oscillatory neural activity and broadband EMG energy—the defining signature of the EMG smearing effect described in Section 1. Whole-component rejection would discard the neural content along with the artifact, whereas ICA-S^3^M leverages the artifact probability to separate the two within each IC, preserving the oscillatory structure while removing the broadband contamination.

### 3.4 Naturalistic data

We next applied both methods to the continuous naturalistic EEG recording. Because no ground-truth clean signal exists in this setting, we evaluated performance using the blind Spectral Power Ratio (SPR), which quantifies the amount of residual high-frequency artifact remaining in the cleaned signal. These results are to demonstrate that ICA-S^3^M operates end-to-end on uncontrolled, naturalistic data—not to establish statistical supremacy over the CNN, which would require a multi-participant naturalistic cohort.

**Figure 13:**
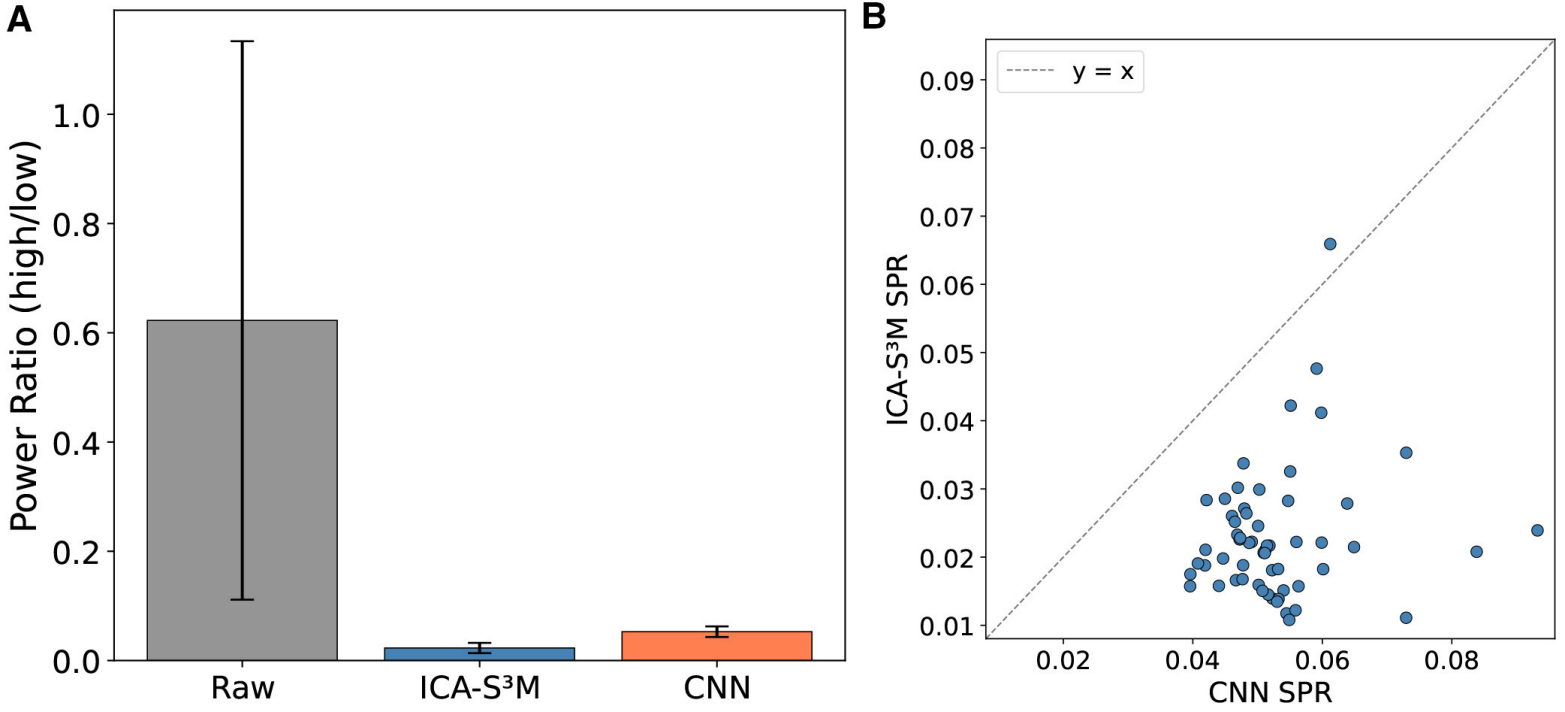
Results for naturalistic data. **(A)** Mean spectral power ratio (SPR) across all 59 EEG channels for the raw (uncleaned), CNN-cleaned, and ICA-S^3^M-cleaned signals. SPR is defined as the ratio of high-frequency power (30–100Hz) to low-frequency power (1–30Hz), where lower values indicate less residual EMG contamination. Both methods substantially reduce SPR relative to raw, with ICA-S^3^M achieving a significantly lower mean SPR than CNN (Wilcoxon signed-rank test, *p* = 2.5 × 10^−11^). **(B)** Per-channel SPR comparison between ICA-S^3^M and CNN. Each point represents one of 59 EEG channels; points below the diagonal indicate channels where ICA-S^3^M achieves a lower (better) SPR than CNN. ICA-S^3^M yields a lower SPR on 58 out of 59 channels, demonstrating consistent better performance in high-frequency artifact suppression across the scalp.

Both methods substantially reduced broadband EMG contamination relative to the uncleaned signal. The CNN reduced the mean SPR from 0.623 ± 0.511 (raw) to 0.053 ± 0.010, representing an approximately 12-fold reduction in the high-to-low frequency power ratio. ICA-S^3^M achieved a further reduction to 0.023 ± 0.009, roughly halving the residual artifact power relative to the CNN. A Wilcoxon signed-rank test across all 59 channels confirmed that ICA-S^3^M produced significantly lower SPR values than the CNN (*W*, *p* = 2.52 × 10^−11^), with ICA-S^3^M yielding a lower SPR on 58 of 59 channels, as shown in Figure 13.

#### 3.4.1 Visual inspection

**Figure 14:**
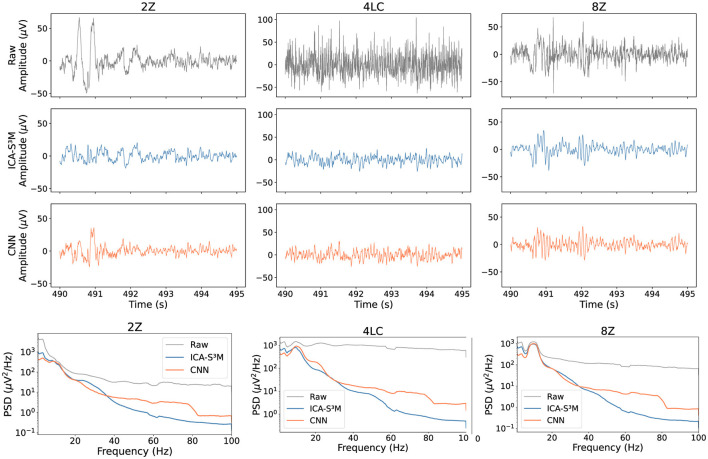
Naturalistic data results for a representative 3s segment. **Top** Time-domain traces showing the raw contaminated signal, and outputs of ICA-S^3^M and the CNN. **Bottom** Power spectral density for 2Z (frontal), 4LC (temporal), and 8Z (occipital).

Visual inspection of the time traces and power spectral densities from the naturalistic recording further confirms that both the CNN and ICA-S^3^M successfully recover the underlying EEG from artifact-contaminated data (Figure 14 top). However, a notable divergence emerges in the spectral domain: the CNN consistently produces a more pronounced alpha peak relative to surrounding frequency bands than either ICA-S^3^M or the raw data. This effect is most apparent at electrode 4LC (Figure 14 bottom), where the raw spectrum exhibits an alpha peak of comparable magnitude to the slow/delta peak. ICA-S^3^M faithfully preserves this feature. In contrast, the CNN amplifies the alpha peak well above the low-frequency components, introducing a spectral emphasis absent from the original recording. This observation is consistent with the alpha hallucination effect identified in the simulation study (Section 3.1). At occipital electrode 8Z (Figure 14 bottom), both methods recover an alpha peak that closely matches the raw spectrum, consistent with a strong veridical occipital alpha rhythm that requires little correction.

## 4 Discussion

Here we showed that ICA-S^3^M can effectively remove EMG artifacts from EEG recordings while preserving underlying neural oscillations. Using a comprehensive study spanning simulations, semi-synthetic real data, and naturalistic recordings, we found that ICA-S^3^M outperforms the CNN across multiple spatial domains, artifact conditions, and performance metrics in both time- and frequency-domains. Crucially, the ICA-S^3^M does not “hallucinate” EEG activity that is not present and is effective at preserving underlying neural oscillations whether artifacts are present or not.

A notable finding from the simulation study is the CNN's tendency to introduce spurious alpha-band peaks in some channels while attenuating genuine alpha activity in others. This “hallucinatory” behavior likely reflects a distributional bias in the training data: the EEGdenoiseNet dataset from which the CNN was trained consists predominantly of resting-state EEG segments with prominent alpha oscillations (Zhang et al., 2021). As a result, the network has learned to explain broadband input variation partly through alpha-band structure, effectively hallucinating alpha peaks where none exist in the true signal. Conversely, when the input already contains strong alpha activity with spectral characteristics that differ from the training distribution, the CNN may suppress rather than preserve it. This failure mode underscores a fundamental limitation of supervised denoising approaches: their outputs are shaped by the statistics of the training corpus, and they may impose learned spectral templates on signals whose generative process differs from that of the training data. ICA-S^3^M avoids this pitfall by fitting physiologically-inspired oscillatory models directly to each recording's own time domain content, adapting to the observed signal at hand rather than relying on population-level templates.

A key advantage of ICA-S^3^M over deep learning approaches is model interpretability. In ICA-S^3^M, every cleaning decision is traceable: for each IC and each time point, the model produces an explicit artifact probability that can be inspected to understand why a segment is being removed. The fitted oscillatory models are likewise interpretable, revealing which spectral components the model has identified as neural signal and chosen to protect. This level of transparency is valuable both for scientific validation and for practical debugging—a researcher can examine individual ICs, verify that artifact probability is high during known contamination periods and low during clean periods, and audit the oscillatory models to ensure physiologically plausible components are being preserved. Deep learning models, by contrast, produce a cleaned output without exposing any intermediate representation of what was identified as artifact versus signal. This opacity makes it impossible to detect and diagnose unexpected behavior or to build confidence in the model's outputs when applied to data outside the training distribution—a common scenario in real-world EEG analysis.

The main advantage of the CNN is computational efficiency. Once trained, deploying the model requires minimal time or computational resources, making it attractive for large-scale or real-time applications. ICA-S^3^M is more computationally demanding, as it fits state-space models to each recording individually. However, this per-recording adaptation is precisely what enables ICA-S^3^M to generalize across diverse EEG datasets without retraining. Overcoming or improving upon the ICA-S^3^M's computational requirements is a tractable problem. In contrast, the CNN's “hallucination” and lack of interpretability are fundamental problems that are not easily addressed in scientific settings where ground-truth labels are unobtainable and are in fact the object of the scientific inquiry.

### 4.1 Limitations

ICA-S^3^M has several practical limitations worth noting.

#### 4.1.1 Offline analysis

ICA-S^3^M cannot be applied to real-time EEG processing. The ICA decomposition requires access to the full recording, making ICA-S^3^M inherently an offline tool. For real-time applications, our original switching state-space model (Stone et al., 2025) provides a viable alternative: it operates on a single EEG channel without requiring a full recording and can be deployed in a streaming context. The choice between the two approaches depends on the application. ICA-S^3^M is particularly well-suited to settings where the underlying oscillatory structure is not known in advance, is weak, transient, or highly variable such as our naturalistic recording example, in which musicians improvised freely and the oscillatory dynamics associated with musical cognition may be complex and subject-specific. In contrast, when the oscillations of interest are well-characterized and relatively stable, as in anesthesia monitoring or oscillation-based brain–computer interfaces such as motor imagery, a causal switching state-space model may be sufficient.

##### 4.1.1.1 Computational cost

Fitting per-IC state-space models is more computationally demanding than running a pre-trained denoising network at inference. A representative 122-channel, 54 s recording (41 retained ICs after PCA whitening at 99.9% variance, six 10 s windows) takes ≈25 minutes wall-clock on a desktop Apple M4 Pro (12 cores, 24 GB unified memory) when run as a single-process pipeline. The per-IC sweep fits are highly parallel and the pipeline is structured to support joblib-based multi-process execution on a compute cluster. The computational cost is therefore a tractable engineering problem rather than a fundamental limitation, but it is a real consideration for very large datasets.

##### 4.1.1.2 Single human rater for the semi-synthetic ground truth

The IC–trial labels that define the semi-synthetic ground truth were generated by a single trained rater; a second rater was not available for the lab cohort. To bound the consequences of this, we trained leave-one-subject-out logistic-regression classifiers on the rater's labels using objective IC features (Appendix 4.2). The classifiers reached pooled κ = 0.61 (REST) and κ = 0.60 (EMG) on held-out subjects with ROC-AUC ≥0.88 in both periods. Two caveats apply to this validation. First, the rater's labeling policy is deliberately asymmetric. Ambiguous ICs default to the conservative negative class in each period, so κ is a lower bound on the rater's consistency relative to an idealized rater that could resolve ambiguity perfectly. Second, the LOSO analysis tests whether the rater's rule is reproducible from objective IC features, which is correlated with but not identical to intra-rater test–retest agreement. The residual disagreement (≈17%–20% of IC × trial cells in held-out subjects) is an upper bound on label noise propagating into the semi-synthetic ground truth.

##### 4.1.1.3 Single-subject naturalistic recording

The naturalistic evaluation (Section 3.4) is restricted to a single subject and is therefore feasibility-demonstrating rather than population-generalizing. The blind-metric (SPR, spectral) comparisons reported there are illustrative and are not subjected to inferential statistics. A multi-subject naturalistic comparison is left for future work.

##### 4.1.1.4 Scope of the validation paradigm

Our preservation analyses quantify how faithfully each method recovers the underlying signal in the time domain (RRMSE, Pearson correlation, SNR improvement) and in the spectral domain (per-band magnitude-squared coherence in δ, θ, α, and β; band-integrated alpha power). They do not include task-evoked features such as event-related potentials or stimulus-locked event-related spectral perturbations, because the present experimental paradigm—eye-closed rest interleaved with cued facial-movement blocks—was designed to put a precisely-timed EMG contamination on top of a known background brain state, not to elicit a time-locked neural response. A purpose-built ERP/ERSP-preservation validation, in a paradigm designed to evoke such responses, would be a natural follow-up in future work.

In summary, ICA-S^3^M offers a principled, interpretable, and generalizable solution to the longstanding challenge of EMG artifact removal in EEG. By combining the spatial separation power of ICA with the temporal dynamics delineation of state-space oscillatory modeling, the method adapts to the dynamical structure of each individual recording without relying on population-level templates or labeled training data. Validated across simulation, semi-synthetic, and naturalistic data, ICA-S^3^M consistently preserves neural oscillations across frequency bands while effectively suppressing muscle artifacts. Its transparent artifact probability estimates and fitted oscillatory models enable researchers to audit and understand every cleaning decision, a property that is increasingly important as EEG is used in more complex, uncontrolled real-world environments. We anticipate that ICA-S^3^M will be a useful tool for researchers and clinicians who require both rigorous artifact removal and scientific accountability in their EEG analyses.

## Data Availability

The raw data supporting the conclusions of this article will be made available by the authors, without undue reservation.
