# Supplementary material for: ICA-S3M: switching state-space model guided automatic EEG artifact removal from independent components
Source: Front Neurosci. 2026 Jul 31;20:1865301. doi: 10.3389/fnins.2026.1865301 (PMC13473372; doi:10.3389/fnins.2026.1865301)
Supplement: Supplementary file 1 [file Supplementary_file_1.pdf]

Supplementary Material

ICA-S^3M: Switching State-Space model guided Automatic EEG Artifact Removal from Independent Components

Tou *et al.*

Appendix

Per-Component Ablation: ICA-only and SSSM-only Comparators

To quantify the contribution of each stage of the ICA-S^3M pipeline, we compared the full pipeline against two ablations on the same 29-participant semi-synthetic dataset described in Section 2.3.2:

ICA-only. Stage 1 only. The recording is decomposed by Extended Infomax ICA, ICs classified as muscle artifacts by ICLabel (Pion-Tonachini et al., 2019) with probability ≥ 0.8 are zeroed out, and the remaining ICs are back-projected to sensor space. This is the canonical ICA / ICLabel-based component-rejection baseline.

SSSM-only. Stage 2 only. The two-sweep switching state-space artifact scrubber is applied directly to each EEG sensor time series, bypassing the ICA decomposition. This isolates the contribution of the within-channel oscillator vs. AR(2) separation.

ICA-S^3M (full). Stages 1 and 2 as described in Section 2.2.

Comparators were evaluated on three representative electrodes: Z2 (frontal, $n = 27$; marked as bad in 2 subjects), RB2 (temporal, $n = 29$), and Z11 (occipital, $n = 29$). These electrodes were chosen to match the regional analysis in Section 3.3.

Aggregate RRMSE

Table S1 reports the mean ± SD relative root-mean-square error across subjects for the full recording, the artifact-injected samples, and the clean (rest-only) samples. ICA-S^3M achieves the lowest full-record and artifact-period RRMSE on all three electrodes. On clean spans the three methods are within ≈ 0.05 RRMSE of each other.

In head-to-head paired comparisons on artifact-period RRMSE, ICA-S^3M beat ICA-only on 76/85 subject-channel pairs (89%) and SSSM-only on 76/85 pairs (89%).

Table S1: Per-subject RRMSE on Z2 (frontal, $n = 27$), Z11 (occipital, $n = 29$), and RB2 (temporal, $n = 29$). Mean ± SD across subjects; lower is better. Best value per column in bold.

Channel	Method	RRMSE full	RRMSE artifact	RRMSE clean
Z2	SSSM-only	0.663 ± 0.255	0.866 ± 0.391	$\mathbf{0.384 \pm 0.173}$
	ICA-only	0.798 ± 0.404	1.058 ± 0.601	0.447 ± 0.165
	**ICA-S^{3}M**	$\mathbf{0.545 \pm 0.168}$	$\mathbf{0.647 \pm 0.227}$	0.430 ± 0.134
Z11	SSSM-only	0.477 ± 0.198	0.585 ± 0.221	0.356 ± 0.206
	ICA-only	0.509 ± 0.237	0.655 ± 0.315	$\mathbf{0.328 \pm 0.184}$
	**ICA-S^{3}M**	$\mathbf{0.445 \pm 0.181}$	$\mathbf{0.492 \pm 0.198}$	0.399 ± 0.172
RB2	SSSM-only	0.734 ± 0.148	0.843 ± 0.144	0.618 ± 0.196
	ICA-only	0.944 ± 0.474	1.212 ± 0.702	0.611 ± 0.217
	**ICA-S^{3}M**	$\mathbf{0.689 \pm 0.144}$	$\mathbf{0.776 \pm 0.166}$	$\mathbf{0.604 \pm 0.142}$

Per-Trial Mixed-Effects Comparison

For each (region × period × metric) and each ordered method pair, we fitted a paired-difference linear mixed model on the per-trial values, $d_{ij} = m_{ij}^{\mathrm{A}} - m_{ij}^{\mathrm{B}}$ with $d_{ij} = \mu + u_i + \varepsilon_{ij}$ ($u_i \sim \mathcal{N}(0, \sigma^2_{\mathrm{subj}})$, $\varepsilon_{ij} \sim \mathcal{N}(0, \sigma^2_{\mathrm{resid}})$), fitted by REML in statsmodels `MixedLM` on the same per-trial inputs used in the main analysis. The Bonferroni family covers 18 cells (3 regions × 2 periods × 3 metrics) × 3 ordered method contrasts = **54 tests**, giving $\alpha = 0.05/54 \approx 9.26 \times 10^{-4}$.

Table S2 reports the ICA-S^{3}M vs. ICA-only and ICA-S^{3}M vs. SSSM-only contrasts during artifact-injected periods. Negative RRMSE differences and positive SNR / correlation differences favor ICA-S^{3}M. ICA-S^{3}M is the better method in every artifact-period (region × metric) cell against both comparators, with 13/18 contrasts surviving Bonferroni correction.

Table S2: Per-trial mixed-effects contrasts during artifact-injected periods. Mean paired difference (95% CI) and Bonferroni significance under $\alpha = 9.26 \times 10^{-4}$. Significant cells in bold.

Region	Metric	ICA-S^{3}M − ICA-only	ICA-S^{3}M − SSSM-only
Frontal	RRMSE	$-\mathbf{0.392}$ ($p = 1.0 \times 10^{-5}$)	-0.157 ($p = 2.2 \times 10^{-3}$)
	SNR (dB)	$+\mathbf{2.50}$ ($p = 8.6 \times 10^{-9}$)	$+\mathbf{1.58}$ ($p = 1.7 \times 10^{-6}$)
	Corr.	$+\mathbf{0.080}$ ($p = 3.2 \times 10^{-8}$)	$+\mathbf{0.114}$ ($p < 10^{-12}$)
Temporal	RRMSE	$-\mathbf{0.362}$ ($p = 4.7 \times 10^{-4}$)	$-\mathbf{0.070}$ ($p = 1.4 \times 10^{-12}$)
	SNR (dB)	$+\mathbf{1.75}$ ($p = 4.4 \times 10^{-4}$)	$+\mathbf{0.81}$ ($p = 2.2 \times 10^{-12}$)
	Corr.	$+0.054$ ($p = 1.7 \times 10^{-2}$)	$+\mathbf{0.113}$ ($p < 10^{-12}$)
Occipital	RRMSE	$-\mathbf{0.110}$ ($p = 1.7 \times 10^{-5}$)	$-\mathbf{0.079}$ ($p = 2.2 \times 10^{-8}$)
	SNR (dB)	$+0.81$ ($p = 1.7 \times 10^{-2}$)	$+\mathbf{1.03}$ ($p = 2.5 \times 10^{-4}$)
	Corr.	$+0.024$ ($p = 9.2 \times 10^{-2}$)	$+\mathbf{0.051}$ ($p = 1.8 \times 10^{-7}$)

Together, these results confirm that both stages contribute materially to ICA-S^{3}M's performance on artifact-period data: removing the ICA stage (SSSM-only) loses spatial separation, removing the within-component switching models (ICA-only) loses the ability to extract neural content from EMG-contaminated mixed components, and only the combined pipeline consistently outperforms each in isolation.

ICs in Semi-Synthetic Real Data

After PCA whitening at 99.9 % variance, the ICA decomposition retained a mean of 59.0(124) independent components per participant (median 59, range $[32, 82]$) across the 29 participants, all of which were forwarded to the Stage 2 switching state-space model.

Rater Consistency Analysis

A second human rater was not available for the lab cohort, so we could not report inter-rater agreement on the manual IC–trial labels that define the semi-synthetic ground truth (Section 2.3.2). To gauge how reproducible the single rater's decisions are, we trained two L_2-regularised logistic-regression classifiers to predict the rater's binary labels from a set of objective IC features and evaluated them under leave-one-subject-out (LOSO) cross-validation across the 29 participants. Because each test fold consists of a subject the classifier has never seen during training, classifier accuracy is a *lower bound* on how internally consistent the rater is across the cohort: any rule the model recovers must have been applied similarly enough across subjects to generalise out-of-sample.

Features and Models

For every (IC $\times$ trial) unit we computed 17 features that summarise three classical IC-screening dimensions:

Spectral (9). Log band powers in δ (1–4 Hz), θ (4–8 Hz), α (8–13 Hz), β (13–30 Hz), low-γ (30–50 Hz), and high-γ (50–100 Hz); a broadband HF ratio $\log(P_{30\text{–}100}/P_{1\text{–}30})$; the slope of $\log_{10}(\text{PSD})$ vs. $\log_{10}(f)$ across 20–100 Hz ($1/f$ flatness); and an α-peak prominence index defined as the log peak PSD in 8–13 Hz minus the log geometric-mean PSD in the immediately flanking 7–8 and 13–14 Hz bands. All PSDs were computed with multitaper over 0.5–100 Hz at a bandwidth of 2 Hz.

Topographic (5). Focality (excess kurtosis of |mixing weights|); an outer-ring edge ratio (mean $|w|$ at electrodes whose names begin `LL` or `RR` divided by mean $|w|$ at all other electrodes); a frontal–occipital asymmetry index using the channel groups from Section 2.3.2; a left–right asymmetry index from electrode-name prefixes (midline `Z`-channels excluded); and an active-channel count (the number of electrodes with $|w| > 2 \times \text{median}(|w|)$). Topographic features are constant across trials for a given IC.

Time-domain transient (3). Log variance, excess (Fisher) kurtosis, and the peak-to-MAD ratio ($\max |y| / \text{MAD}(y)$) of the IC source segment.

Each period (REST: clean vs. not-clean; EMG: clear-artifact vs. not) was modelled with an independent L_2-regularised logistic regression (`sklearn.linear_model.LogisticRegression`, $C = 1$, `class_weight = 'balanced'`, `solver = 'lbfgs'`). Features were z-scored within each training fold so that standardisation used training-set statistics only. Evaluation was LOSO (`LeaveOneGroupOut`,

groups = subject ID, 29 folds); all metrics reported below come from the out-of-sample predictions on the held-out subject in each fold. Standardised feature weights were aggregated across the 29 LOSO fits as mean ± SD, so that the weight SDs index how stable each feature's contribution is across the population.

Classifier Performance

Table S3 reports pooled and per-subject performance. Both models reach $\kappa \approx 0.60$ — substantial agreement under Landis and Koch (1977) — on held-out subjects, with ROC-AUC ≥ 0.88 in both periods. No subject collapses to chance (minimum AUC was 0.82 for REST and 0.74 for EMG). Figure S1 shows the pooled confusion matrices.

Table S3: Rater-consistency classifier performance, leave-one-subject-out across the 29 lab participants. *Pooled* = metric computed on the concatenation of held-out predictions; *per-subject* = mean ± SD across the 29 LOSO test folds. Positive class is "not cleanly clean" for REST and "clearly artifact only" for EMG.

	REST		EMG	
	pooled	per-subject	pooled	per-subject
Cohen's κ	0.614	0.575 ± 0.102	0.595	0.568 ± 0.112
ROC-AUC	0.928	0.925 ± 0.033	0.884	0.891 ± 0.052
Balanced accuracy	0.847	—	0.801	—
MCC	0.625	—	0.596	—
N (IC $\times$ trial)	21,321	—	16,528	—
Positive-class base rate	19.9%	—	41.0%	—
Min. per-subject κ	—	0.36	—	0.31
Max. per-subject κ	—	0.79	—	0.80

What the Rater Was Using

Table S4 reports the top-eight standardised features for each period, sorted by |mean weight|. The dominant contribution in both periods is `log_p_highgamma` (REST +2.30±0.12; EMG +2.11±0.08), with offsetting negative weights on β power, α power, and the HF ratio. This is a redistribution of the same broadband signal across collinear features under the L_2 penalty; the directional reading is that the classifier latches onto broadband high-frequency power that is disproportionate to the neural rhythm in the same component, which is the canonical EMG / muscle screening criterion. The REST model additionally relies on time-domain transient features (`peak_mad` +2.19±0.11, `kurtosis` $+1.51 \pm 0.21$), capturing brief blink / movement events that do not broaden the average spectrum. The EMG model is the only one in which a topographic feature survives at high weight: `fp_asym` ($+0.62 \pm 0.02$), favoring frontally biased ICs, consistent with the facial-EMG injection paradigm. Across both models, the LOSO weight standard deviations are tight (≤ 0.22 in standardized units for every feature in either top-eight), so the rater is applying substantially the same rule across the 29 subjects rather than a per-subject heuristic.

Table S4: Top eight standardised feature weights per model (mean ± SD across the 29 LOSO folds), sorted by |mean weight|. Positive weights push the IC–trial toward the positive class ("not cleanly clean" for REST; "clearly artifact only" for EMG).

REST			EMG		
feature	weight	SD	feature	weight	SD
`log_p_highgamma`	+2.295	0.123	`log_p_highgamma`	+2.112	0.082
`peak_mad`	+2.189	0.109	`log_p_beta`	−1.666	0.050
`log_p_beta`	−1.862	0.059	`hf_ratio`	−1.646	0.072
`hf_ratio`	−1.739	0.109	`log_p_alpha`	−1.212	0.030
`kurtosis`	+1.512	0.214	`log_p_lowgamma`	+0.801	0.067
`log_p_alpha`	−1.220	0.037	`log_p_theta`	−0.798	0.018
`log_p_lowgamma`	+0.713	0.078	`log_p_delta`	−0.752	0.030
`log_p_theta`	−0.498	0.029	`fp_asym`	+0.618	0.016

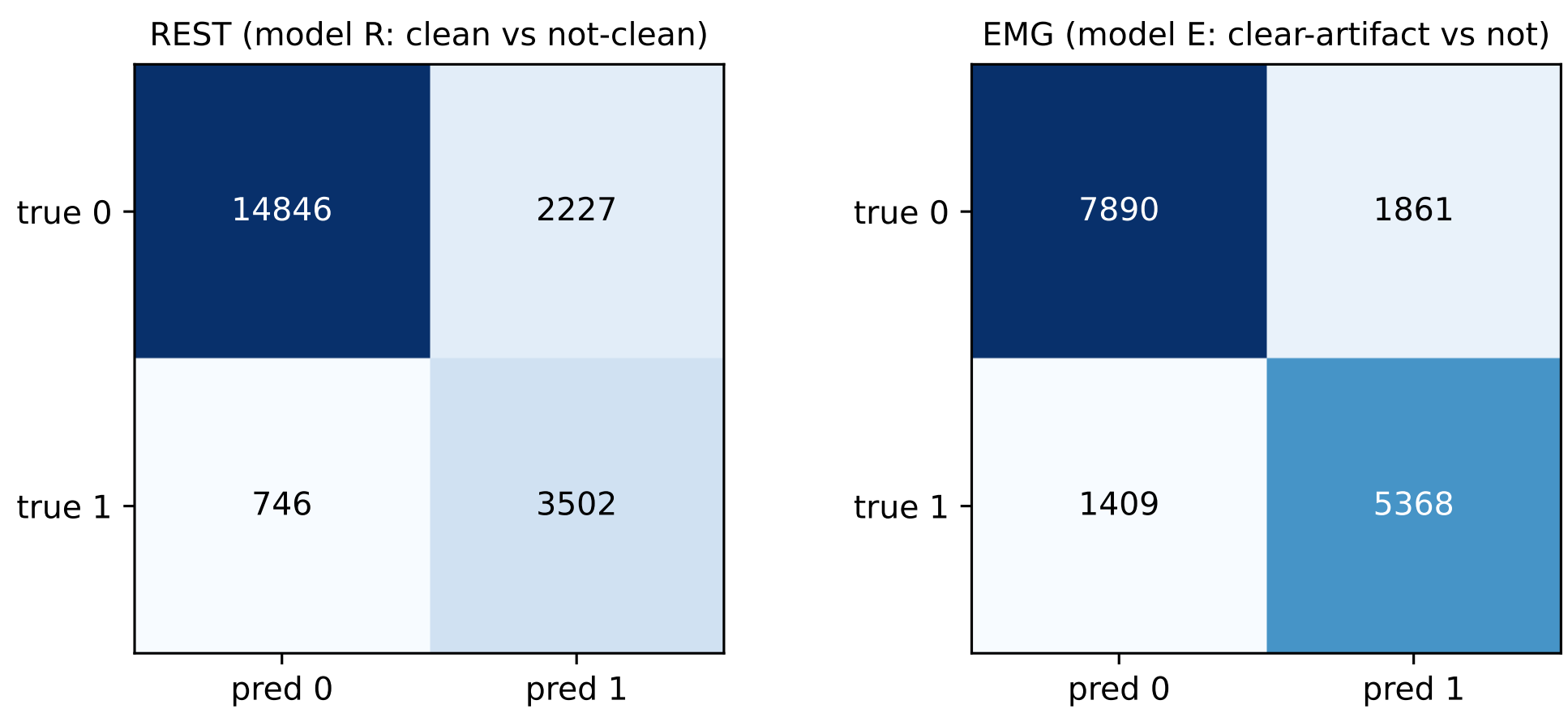

Figure S1: Pooled confusion matrices for the leave-one-subject-out rater-consistency classifiers (rows = manual label, columns = classifier prediction). Left: REST period (positive class is "not cleanly clean"). Right: EMG period (positive class is "clearly artifact only").

References

Landis, J. R. and Koch, G. G. (1977). The measurement of observer agreement for categorical data. *Biometrics*, 33(1):159–174.

Pion-Tonachini, L., Kreutz-Delgado, K., and Makeig, S. (2019). ICLabel: An automated electroencephalographic independent component classifier, dataset, and website. *NeuroImage*, 198:181–197.

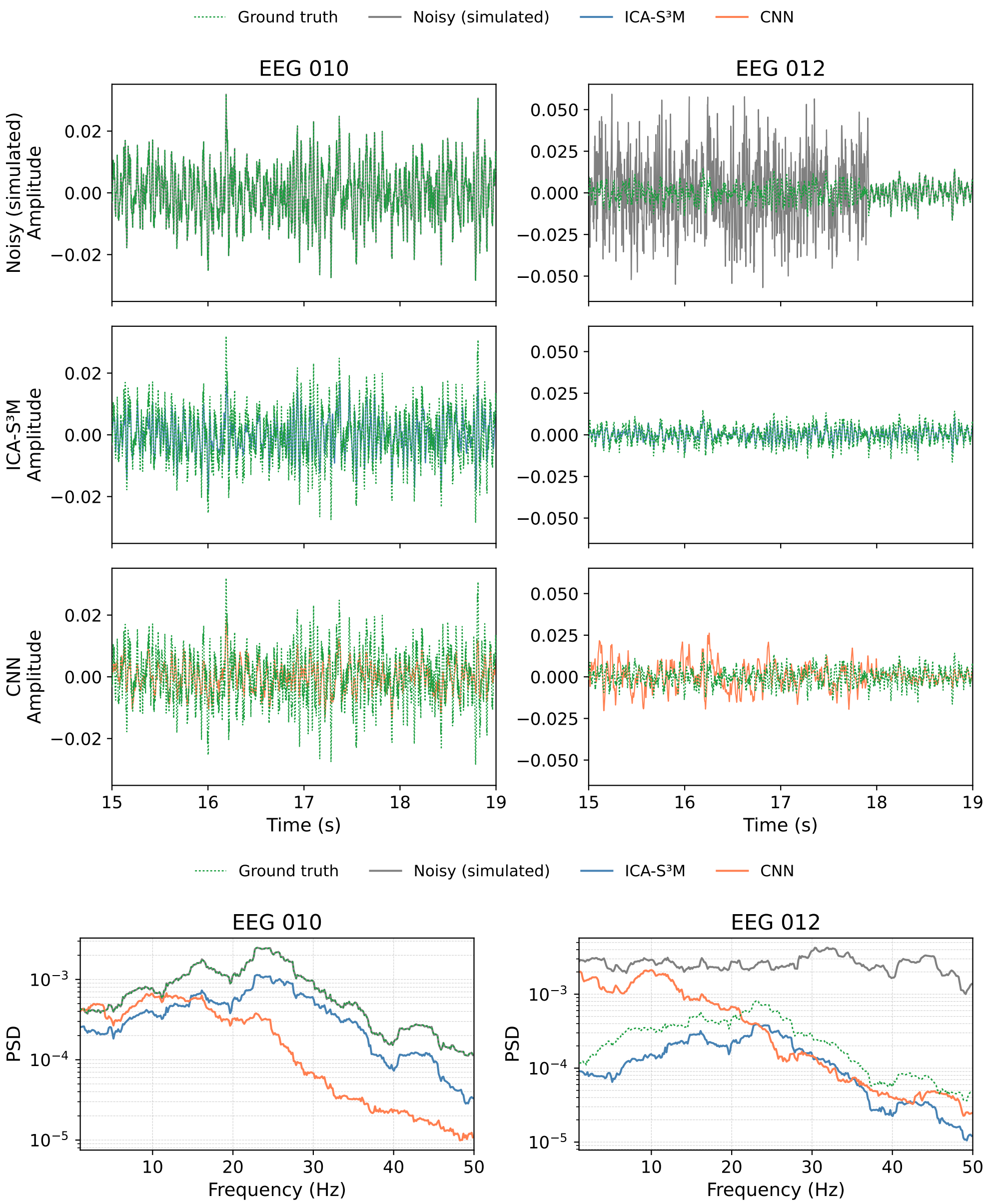

Figure S2: Simulation results for a representative 3 s segment when ground truth alpha is absent. **Top:** Time-domain traces showing the ground truth (GT), the artifact-contaminated signal (Noisy), and the reconstructions produced by ICA-S^{3}M and CNN. **Bottom:** Power spectral densities for two example electrodes. At both electrodes, CNN "hallucinates" an alpha peak that is absent from the ground truth.
